# The pathological mechanisms and potential therapeutic strategies for renal anemia

**DOI:** 10.3389/fphar.2026.1847564

**Published:** 2026-08-25

**Authors:** Jia-Xing Rao, Hao-Ming Zhou, Kim Fey Leu, Ming Liu, Ze-Feng Liu, Yu-Ping Tang

**Affiliations:** 1 Key Laboratory of Shaanxi Administration of Traditional Chinese Medicine for TCM Compatibility, Shaanxi University of Chinese Medicine, Xianyang, China; 2 MK Faculty of Medicine and Health Sciences, Universiti Tunku Abdul Rahman Jalan Sungai Long, Selangor, Malaysia; 3 White Heron Pharmaceutical Sdn Bhd, Kuala Lumpur, Malaysia

**Keywords:** erythropoietin, natural products, pathological mechanisms, renal anemia, therapeutic strategies

## Abstract

Renal anemia (RA) is a common complication associated with chronic kidney diseases. Its pathological mechanism is attributed to the combined effects of insufficient production of erythropoietin by the kidneys, iron metabolism disorders, and chronic inflammatory conditions. The main pillars of medicine treatment for these aspects include erythropoiesis-stimulating agents, iron supplementation, and novel hypoxia-inducible factor prolyl hydroxylase inhibitors. Traditional Chinese medicine shows potential advantages across multiple targets in improving anemia and renal function in patients with RA. This review aims to systematically explain the current pathophysiological understanding of RA, examine the prevailing treatment approaches, and summarize the pharmacological mechanisms and research findings related to natural products and their active compounds used in RA treatment. This review is to provide new insights and references to support future in-depth research and clinical management of RA.

## 1 Introduction

Renal anemia (RA) is a prevalent complication of chronic kidney disease (CKD), particularly in advanced stages, in which it manifests with debilitating symptoms that substantially impair patients’ quality of life. This pathophysiological connection underscores the importance of RA management as a critical therapeutic priority in modern nephrology (Fishbane and Coyne, 2020). The etiology of RA is multifactorial, encompassing insufficient erythropoietin (EPO) synthesis, impaired hypoxia-inducible factor (HIF) signaling, iron metabolism dysregulation, chronic inflammation, and reduced red blood cell (RBC) survival. Among these, absolute or relative EPO deficiency is recognized as the primary pathogenic mechanism (Mimura et al., 2015). HIF, a transcription factor activated under hypoxic conditions, plays a crucial role in stimulating EPO production in response to tissue hypoxia during anemia, thereby promoting erythropoiesis and regulating iron metabolism (Hu et al., 2021). Furthermore, nephropathy is often accompanied by iron metabolism disorders that affect iron absorption and utilization, thereby exacerbating anemia symptoms (Wang X. et al., 2023). In addition, other factors, including uremic toxins, chronic inflammation, and shortened erythrocyte survival, contribute to anemia. Therefore, the pathophysiology of RA is complex and multifactorial, with the interplay among these components being increasingly elucidated through systematic integration of clinical and experimental data.

A systematic literature search was performed in the following five electronic databases: PubMed, Web of Science, Embase, Medline, and Google Scholar. Keywords were used in searching, including “renal anemia,” “chronic kidney disease,” “mechanisms,” “pathological mechanism,” “erythropoietin,” “hypoxia-inducible factor,” “iron metabolism,” “chronic inflammatory,” “therapeutic drugs,” “treatment strategy,” “traditional Chinese medicine,” “Chinese herbal medicine,” “monomers,” “extracts,” “natural product,” and “traditional preparations.” The inclusion criteria for this review encompassed studies on the pathologic mechanisms and pathogenesis of RA, as well as research on conventional treatments, including erythropoiesis-stimulating agents (ESAs), HIF prolyl hydroxylase inhibitors, iron therapy, and blood transfusion, and research on traditional Chinese medicine (TCM) treatments for RA, including extracts, active monomers, and traditional preparations. All published studies, including *in vitro* experiments, *in vivo* experiments, and clinical trials, were included without language restrictions. Conference papers, case reports, studies with incomplete data, experience sharing articles, editorials, and posters were excluded. This review followed the Preferred Reporting Items for Systematic Reviews and Meta-Analyses (PRISMA) guidelines.

Currently, the cornerstone of RA management rests on a triad of conventional therapies: ESAs, intravenous or oral iron supplementation, and RBC transfusion in acute settings. While effective, this paradigm faces significant challenges, including cardiovascular risks associated with high-dose ESA use, functional and absolute iron deficiencies, and the inherent risks of transfusion therapy. The recent advent of hypoxia-inducible factor prolyl hydroxylase inhibitors represents a paradigm shift, offering a novel oral therapeutic alternative. By inhibiting prolyl hydroxylase enzymes, these agents stabilize HIF-α subunits, thereby physiologically stimulating endogenous EPO synthesis and concomitantly enhancing iron mobilization and bioavailability (Heras-Benito, 2023). In TCM theory, the pathogenesis of RA is fundamentally rooted in deficiency patterns, primarily involving the spleen and kidney. From a pathophysiological perspective, dysfunction of these two organ systems impairs digestive and absorptive functions required to provide hematopoietic substrates, while also disrupting the endocrine and metabolic activities essential for EPO production and bone marrow regulation. This multifactorial disturbance ultimately leads to impaired RBC generation, manifesting clinically as anemia (Li Y. et al., 2023; Fang H. J. et al., 2024). Modern research suggests that this TCM-based etiology may be associated with disturbances in erythropoiesis, iron metabolism, and the chronic inflammatory state characteristic of RA. Accordingly, TCM therapeutic strategies focus on reinforcing the kidney and spleen to promote hematopoiesis and restore systemic homeostasis (Zhang K. X. et al., 2025). An increasing number of studies have shown that the monomers, extracts and preparations derived from TCM have potential effects in treating RA, including astragalus polysaccharide, astragaloside IV (Liang et al., 2023), Angelica sinensis polysaccharide (Shen et al., 2024), Danggui Buxue decoction (Ma et al., 2022), Jianpi-Yishen decoction (Huang H. P. et al., 2024), Shengxuening (Zeng et al., 2020), and Shengxuebao mixture (Yu et al., 2025). Their mechanisms include stimulating endogenous EPO production, improving iron utilization, modulating key inflammatory pathways, and enhancing nutrient absorption. These actions synergistically promote erythropoiesis and elevate hemoglobin (Hb) levels. Future therapeutic strategies will focus on the precise regulation of HIF dynamic balance, the development of oral small-molecule ESA analogues, the exploration of EPO gene-targeted delivery systems, and the clinical application of iron regulatory protein antagonists. Drawing upon relevant literature published in recent years, this article comprehensively examines the multifaceted pathological mechanisms and standard treatment options for RA, with a specific focus on elucidating the emerging role and potential mechanisms of TCM.

## 2 The pathological complexity of RA

The pathogenesis of RA involves multiple interconnected mechanisms that collectively contribute to disease progression. A critical factor is the relative or absolute deficiency of EPO arising from progressive loss of functional renal EPO-producing cells (REPCs) in the injured kidney interstitium, which directly impairs erythropoiesis. Concurrent impairment of HIF signaling compromises cellular adaptation to hypoxic conditions, disrupting the transcriptional activation of EPO and other hypoxia-responsive genes essential for erythropoiesis and iron metabolism. Dysregulated iron metabolism, characterized by elevated hepcidin levels and subsequent functional iron deficiency, further restricts the availability of iron for Hb synthesis, thereby exacerbating anemia. Additional contributing factors, including chronic inflammation, oxidative stress, mitochondrial dysfunction, and uremic toxin accumulation, play critical roles in initiating and sustaining the disease process.

### 2.1 EPO deficiency

**FIGURE 1:**
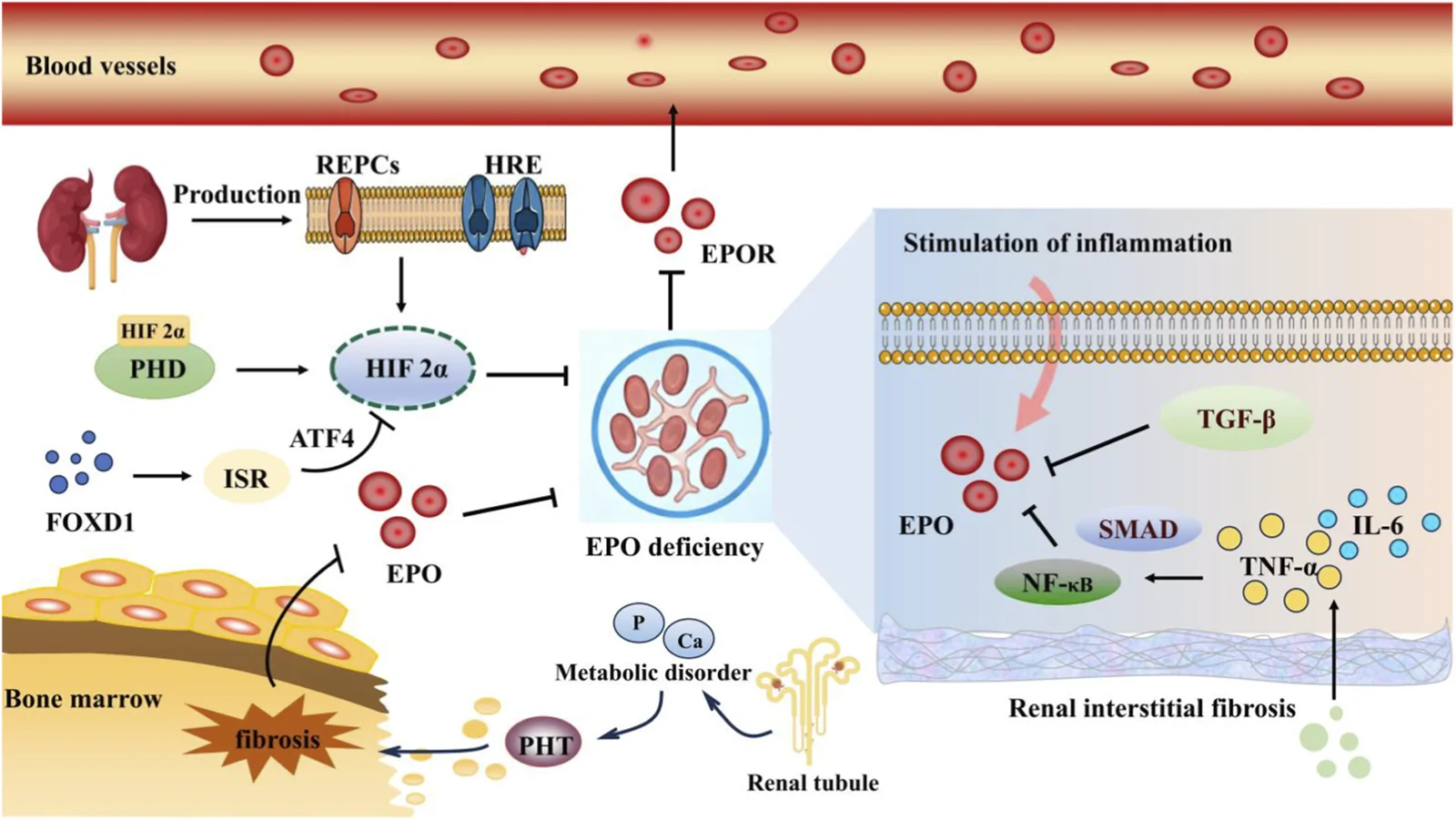
The pathological mechanism of EPO-induced RA.

EPO is a glycoprotein hormone secreted by REPCs in the proximal tubular interstitium of the kidney and is tightly regulated by hypoxia to maintain tissue oxygen homeostasis. As the key molecular regulator of erythropoiesis, EPO has consistently been recognized as a core pathogenic element in RA, and its biosynthesis and metabolic balance are precisely controlled by hypoxic signals, inflammatory microenvironments, and hormonal networks (Figure 1) (Souma et al., 2015). The HIF-PHD axis, which serves as the principal oxygen-sensing mechanism governing EPO transcription, is discussed in detail in Section 2.2 (HIF deficiency).

Chronic inflammation disrupts this oxygen-sensing machinery by directly impairing HIF-α stability and transcriptional activity through Nuclear factor-*κ*B (NF-*κ*B) activation, thereby reducing EPO transcription (Matsuoka et al., 2024). Beyond this acute suppression, sustained inflammation drives REPC-to-myofibroblast transdifferentiation and mitochondrial dysfunction through activation of SMAD and NF-*κ*B signaling by transforming growth factor-β (TGF-β) and Tumor necrosis factor-α (TNF-α), ultimately resulting in irreversible loss of EPO biosynthetic capacity (Lanser et al., 2021).

In addition to these intrinsic renal mechanisms, systemic endocrine abnormalities further contribute to EPO deficiency. In patients with progressive CKD, decreased glomerular filtration rate and impaired tubular function disrupt calcium-phosphorus homeostasis, which in turn may affect the endocrine regulation of erythropoiesis (Marando et al., 2024). Impaired tubular reabsorption of calcium and calcitriol deficiency due to reduced 1-α-hydroxylase activity culminate in hypocalcemia, which in turn drives sustained parathyroid hormone (PTH) hypersecretion and the development of secondary hyperparathyroidism (SHPT) (Tsai et al., 2024). Elevated PTH levels promote myelofibrosis, damaging the bone marrow microenvironment and consequently suppressing hematopoietic activity. Moreover, PTH exacerbates RA by reducing EPO production, attenuating peripheral EPO sensitivity, and shortening erythrocyte lifespan (Tanaka et al., 2018).

Reduced EPO biosynthesis by REPCs thus constitutes a critical factor in RA pathogenesis. Under physiological conditions, REPC-derived EPO binds to EPORs on erythroid progenitors, thereby suppressing apoptosis and promoting proliferation and differentiation (Suzuki and Yamamoto, 2016). However, in RA, this regulatory axis is disrupted at multiple levels. EPO biosynthesis is compromised by loss and transdifferentiation of REPCs, whereas EPOR expression on erythroid progenitors is simultaneously downregulated by inflammatory cytokines and circulating uremic toxins (Yuan et al., 2024). As RA progresses with renal vascular injury or interstitial fibrosis, progressive loss of functional REPCs results in an irreversible deficit in EPO synthetic capacity.

### 2.2 HIF deficiency

HIF, an oxygen-sensitive transcription factor, regulates cellular adaptation to hypoxia. It orchestrates critical adaptive responses, including vascular remodeling, RBC production, and metabolic reprogramming (Nakanishi and Kuragano, 2024). HIF was first discovered in 1992 as a heterodimer composed of alpha and beta subunits. The alpha subunit serves as the primary oxygen-regulated component, with three isoforms subsequently identified, designated HIF-1α, HIF-2α, and HIF-3α (Tanaka, 2017). HIF-1α and HIF-2α bind to DNA sequences known as hypoxia response elements (HREs), which activate the expression of target genes. In contrast, HIF-3α lacks a DNA-binding domain and negatively modulates hypoxia-induced cellular responses (Yousaf and Spinowitz, 2016). Despite structural similarities, HIF-α isoforms exhibit distinct tissue-specific expression and functional divergence. HIF-1α is ubiquitously expressed and particularly abundant in renal tubular epithelial cells, where it promotes vascular endothelial growth factor (VEGF) transcription (Zhang et al., 2024). HIF-2α exhibits a restricted expression pattern; it is enriched in renal mesenchymal stromal cells and serves as the master transcriptional regulator of EPO (Li Z. L. et al., 2022). HIF-2α is the predominant renal isoform driving EPO production, whereas HIF-1α plays no direct role in EPO transcription. Within the renal microenvironment, HIF-2α sustains erythropoiesis, while HIF-1α promotes fibrogenesis and inflammation. The REPC-to-myofibroblast transition in RA favors HIF-1α, exacerbating fibrosis and EPO suppression (Solak et al., 2016). This functional dominance of HIF-2α in EPO regulation underpins the therapeutic rationale for PHD inhibitors.

Cellular HIF levels are primarily governed by oxygen-dependent degradation, with PHD enzymes serving as rate-limiting regulators. Following hydroxylation, the von Hippel-Lindau (VHL) E3 ubiquitin ligase recognizes HIF-α, leading to its ubiquitination and subsequent proteasomal degradation, thereby suppressing EPO gene expression and promoting anemia pathogenesis (Gilreath and Rodgers, 2020; Tanaka, 2016; Liao et al., 2024). During hypoxia or pharmacological PHD inhibition, PHD activity is suppressed, leading to HIF-α stabilization, nuclear translocation, and dimerization with HIF-β, which in turn activate hypoxia-responsive genes (Sato et al., 2019; Sato and Takeda, 2023). In the context of EPO regulation, this process is mediated primarily through HIF-2α stabilization, as HIF-1α does not contribute to EPO transcription. In RA, this oxygen-sensing machinery is disrupted not only by inflammatory cytokines that destabilize HIF-α but also by epigenetic modifications that render HIF target genes transcriptionally refractory (Haase, 2017; Thévenod et al., 2022).

**FIGURE 2:**
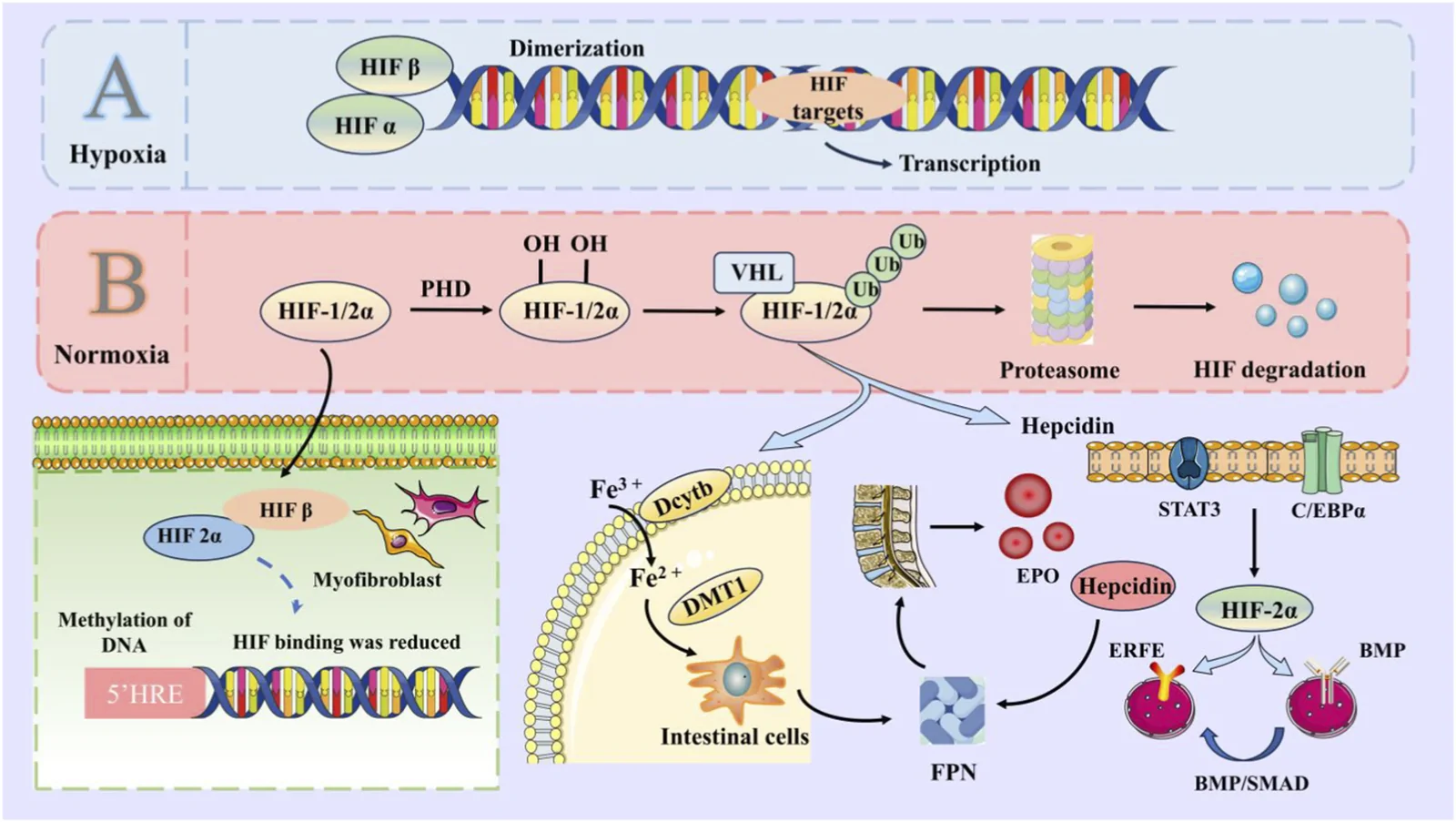
Pathological mechanisms of RA associated with HIF deficiency. **(A)** Under hypoxic conditions, HIF-α/HIF-β heterodimers bind to HREs in the EPO promoter, thereby activating its expression. **(B)** Under normoxic conditions, PHD-mediated hydroxylation of HIF-2α targets it for VHL-dependent ubiquitination and subsequent proteasomal degradation, thereby suppressing EPO transcription.

The HIF-PHD axis drives RA pathogenesis through multidimensional mechanisms that involve impaired EPO biosynthesis and dysregulated iron metabolism. In REPCs, HIF-2α stabilization under hypoxia or PHD inhibitor intervention enables binding to the EPO gene enhancer (5′-TACGTGCT-3′), thereby activating EPO transcription and promoting Hb synthesis (Figure 2) (Dahl et al., 2022). In the pathological progression of RA, REPC-to-myofibroblast transdifferentiation imposes epigenetic barriers that impede EPO transcription. Regulatory elements in the EPO locus undergo DNA hypermethylation due to elevated DNA methyltransferase activity, alongside repressive histone modifications and increased nucleosome occupancy at the HIF-binding site, which collectively suppress the transcriptional activation of EPO (Shih et al., 2018). This chromatin-mediated resistance to HIF renders the EPO gene inaccessible to the transcriptional machinery, even when HIF-2α is stabilized, explaining the limited efficacy of PHD inhibitors in advanced fibrotic CKD.

Beyond EPO regulation, HIF signaling critically modulates iron metabolism. In the duodenum, dietary Fe^3+^ is reduced to Fe^2+^ by duodenal cytochrome B (Dcytb) and subsequently imported via Divalent metal transporter 1 (DMT1), then exported to the circulation through ferroportin (FPN), which is expressed on enterocytes, hepatocytes, and macrophages (Li J. et al., 2023; Hasegawa et al., 2018). HIF-1α directly suppresses hepcidin transcription by binding to HREs in the hepcidin promoter, competing with signal transducer and activator of transcription 3 (STAT3) and C/EBPα, while HIF-2α promotes erythroferrone (ERFE) expression in erythroblasts to antagonize the BMP/SMAD pathway that drives hepcidin production, thereby establishing a physiologically coupled erythropoiesis-iron utilization loop (Li Z. L. et al., 2022; Sanghani and Haase, 2019). Collectively, these observations establish HIF-2α dysfunction as a nexus linking impaired EPO production, disrupted iron metabolism, and maladaptive metabolic reprogramming—the tripartite failure central to RA pathogenesis (Semenza, 2023; Haase, 2021).

### 2.3 Disorders of iron metabolism

Iron is essential for Hb synthesis, redox reactions, and cellular proliferation (Nakanishi et al., 2020). Systemic iron homeostasis is orchestrated by the hepcidin-ferroportin axis, which coordinates crosstalk among hepatic, enteric, and hematopoietic compartments (Nakanishi et al., 2016). Dietary iron absorbed by duodenal enterocytes is exported via FPN1, bound to transferrin, and delivered to erythroid progenitors for EPO-driven heme synthesis (Ogawa et al., 2023). Senescent erythrocytes undergo phagocytosis by splenic macrophages, and the liberated iron is subsequently salvaged and recycled via the reticuloendothelial system, thereby completing the closed-loop circuit (Li C. H. et al., 2022; Lee et al., 2021).

In RA, systemic iron homeostasis is disrupted at multiple levels. Renal failure impairs hepcidin clearance, while chronic inflammation drives its overproduction predominantly via the IL-6/JAK2/STAT3 pathway. This dysregulation leads to a state of combined iron deficiency, defined as the simultaneous presence of absolute iron deficiency with depleted stores alongside functional iron deficiency with impaired mobilization (Nemeth and Ganz, 2023; Borawski et al., 2021). By promoting FPN1 degradation, hepcidin blocks intestinal iron absorption and macrophage iron release, limiting the iron supply to erythroid progenitors and presenting as low transferrin saturation with normal or elevated ferritin. Beyond iron sequestration, hepcidin downregulates EPOR expression on erythroid progenitors via JAK2/STAT5 pathway inhibition, thereby directly attenuating EPO responsiveness (Wang X. et al., 2023). ERFE, produced by erythroblasts in response to EPO, normally provides negative feedback by sequestering BMP ligands and antagonizing hepatocyte hepcidin transcription (Nakanishi et al., 2019). In RA, insufficient EPO production leads to inadequate ERFE induction, resulting in persistent elevation of serum hepcidin levels and perpetuation of the iron-restricted state.

At the cellular level, the iron-regulatory protein/iron response element (IRP-IRE) system post-transcriptionally regulates transferrin receptor 1 (TfR1), DMT1, ferritin, and FPN in response to intracellular iron levels (Li C. H. et al., 2022). Under physiological conditions, low iron stabilizes TfR1 and DMT1 mRNAs to enhance uptake while repressing ferritin and FPN translation to limit storage and efflux. In RA, however, this finely tuned system is overridden by inflammatory signals, locking macrophages and intestinal epithelial cells in an iron-retentive state that perpetuates functional iron deficiency despite systemic signals that should promote mobilization (Matsuoka et al., 2024).

A critical consequence of iron dysregulation is ferroptosis, an iron-dependent form of regulated cell death driven by lipid peroxide accumulation (Li L. et al., 2025). In REPCs and proximal tubular epithelial cells (PTECs), ferroportin deficiency and hepcidin excess promote intracellular iron overload, sensitizing these cells to ferroptotic death. Ferroptosis of PTECs disrupts the renal microenvironment that supports REPC function, while ferroptotic REPCs directly deplete the cellular source of EPO. Ferroptotic cells release DAMPs that perpetuate local inflammation, and iron liberated from dying cells amplifies oxidative damage in adjacent REPCs, establishing a self-perpetuating cycle of REPC loss and EPO deficiency (Soofi et al., 2024; Li L. et al., 2025). Mitochondrial dysfunction is central to this process: these organelles are major consumers of iron for heme and iron-sulfur cluster biosynthesis yet also primary sites of ROS generation. Perturbation of iron-sulfur cluster homeostasis leads to respiratory chain dysfunction and iron accumulation, increasing ferroptosis susceptibility (Lu et al., 2025). In REPCs, this mitochondrial dysfunction not only triggers ferroptosis but also activates the integrated stress response, which directly suppresses HIF-2α target genes including EPO (Matsuoka et al., 2024). The hepcidin-ferroptosis relationship thus constitutes a self-amplifying loop: excess hepcidin promotes ferroportin degradation and intracellular iron overload, sensitizing cells to ferroptosis (Scindia, 2024), while ferroptotic cell death releases iron-laden debris and DAMPs that further stimulate hepcidin production.

**FIGURE 3:**
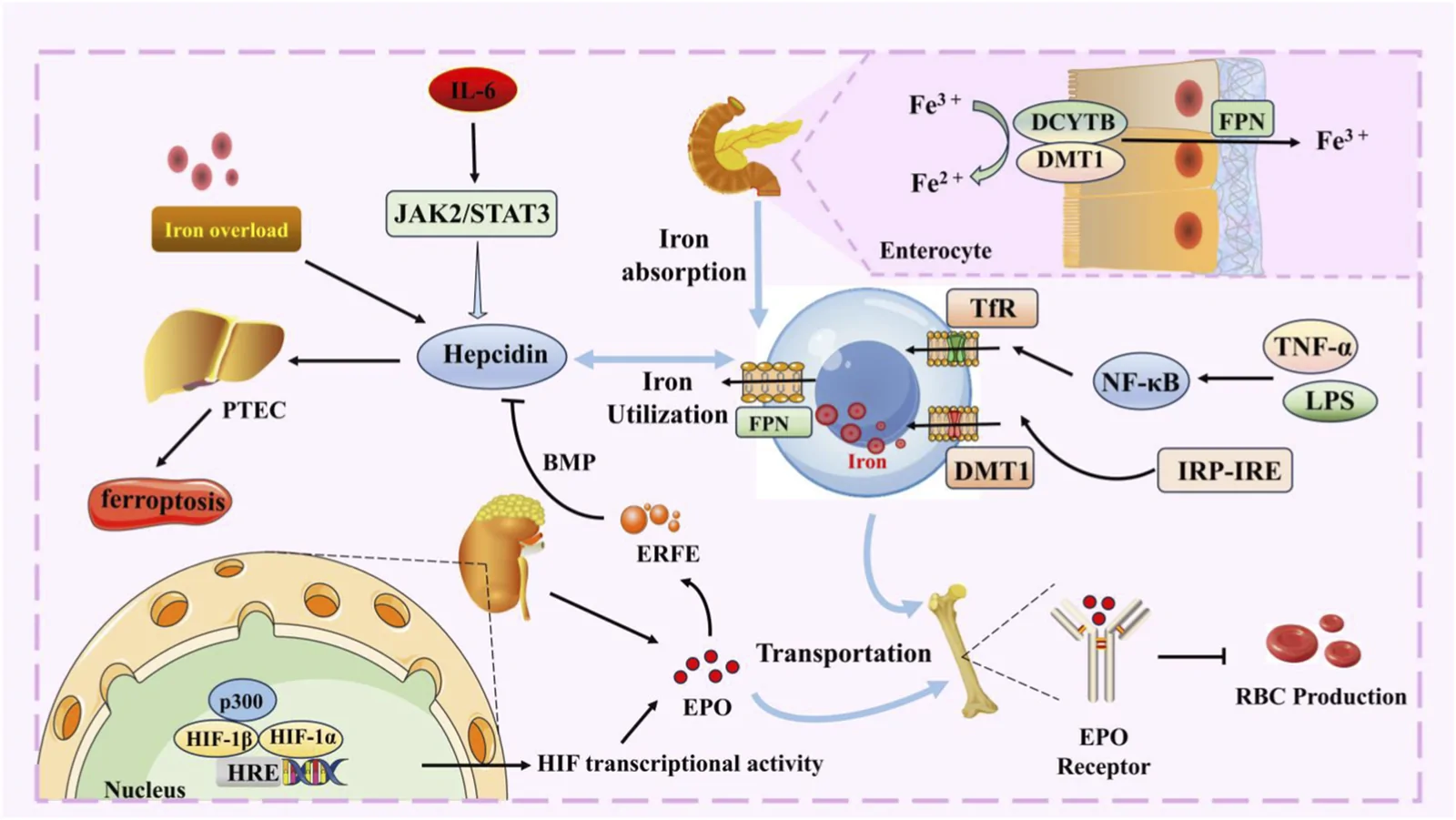
Mechanism of RA induced by disturbance of iron metabolism.

While iron supplementation corrects absolute deficiency, intravenous iron can transiently exceed transferrin-binding capacity, generating non-transferrin-bound iron that catalyzes Fenton reaction-mediated ROS production. This labile iron is preferentially taken up by REPCs and tubular cells via ZIP14 and L-type calcium channels, inducing lipid peroxidation, mitochondrial damage, and impaired EPO synthesis (Matsuoka et al., 2024). Therefore, iron metabolism disorder in RA manifests as a pathological cascade in which excessive hepcidin drives systemic iron restriction, cellular iron overload, and subsequent ferroptosis. Ferroptotic death of REPCs directly depletes the cellular source of EPO, while concomitant ferroptosis of tubular epithelial cells disrupts the renal microenvironment required for REPC survival (Figure 3). Ultimately, this cascade converges on REPC loss and EPO deficiency, establishing ferroptosis as a key mechanistic link between iron dysregulation and impaired erythropoiesis in RA (Hu et al., 2026).

### 2.4 Inflammatory response

Inflammation constitutes a major pathogenic driver in RA, exerting a multifaceted impact on erythropoiesis. Specifically, it suppresses renal EPO production, disrupts systemic iron metabolism, and inhibits the proliferative activity of erythroid progenitors in the bone marrow (Solak et al., 2016). A chronic inflammatory environment, characterized by uremic toxin accumulation, oxidative stress, and comorbidities, is a hallmark of CKD. This environment sustains a self-perpetuating cycle that underlies ESA hyporesponsiveness and refractory anemia. This section explores the convergent inflammatory pathways that establish the erythropoietic “inflammatory blockade,” with a focus on emerging mechanisms such as inflammasome activation, pyroptosis, and suppressors of cytokine signaling (SOCS)-mediated feedback dysregulation.

Chronic inflammation disrupts REPC function through multiple mechanisms. NF-*κ*B activated by pro-inflammatory cytokines directly suppresses HIF-α stability and transcriptional activity, reducing EPO synthesis. Concurrently, TGF-β and TNF-α, via SMAD and NF-*κ*B signaling, drive REPC-to-myofibroblast transdifferentiation, a process accompanied by metabolic reprogramming and mitochondrial dysfunction. REPCs, derived from the FOXD1 lineage, are normally quiescent perivascular fibroblasts; under pathological conditions, they gain proliferative capacity at the expense of EPO-producing function. Mitochondrial impairment activates the integrated stress response (ISR), and ATF4-mediated reprogramming suppresses HIF-2α target genes including EPO (Kobayashi and Haase, 2024). Single-cell RNA sequencing of TFAM-deficient interstitial cells confirmed upregulation of ISR-related genes and downregulation of EPO transcription, directly linking mitochondrial health to EPO production capacity. This transdifferentiation results in irreversible loss of EPO biosynthetic capacity (Gluba-Brzózka et al., 2020; Kaneko et al., 2022). Beyond cellular remodeling, NF-*κ*B activation disrupts HIF-2α stability and DNA binding, blocking hypoxic EPO induction even when the oxygen-sensing pathway remains intact (Matsuoka et al., 2024).

In the bone marrow, inflammation suppresses erythropoiesis through both cell-autonomous and microenvironmental mechanisms. TNF-α and interferon-gamma (IFN-γ) directly inhibit erythroid progenitor proliferation and induce apoptosis via caspase activation and Fas upregulation (Yan and Xu, 2020). Inflammatory cytokines also reshape the marrow niche by inducing stromal cells to secrete IL-8 and additional TNF-α, promoting fibrosis and abnormal angiogenesis that disrupts the supportive architecture for erythroid development (Gluba-Brzózka et al., 2020). Chemokine upregulation, particularly C-X-C motif chemokine ligand 10, impairs progenitor cell mobilization and homing. These combined effects, including direct progenitor suppression and a compromised microenvironment, underlie bone marrow resistance to EPO stimulation, the fundamental cause of ESA hyporesponsiveness.

The IL-6/JAK/STAT3 pathway serves as the critical hub linking inflammation to systemic iron dysregulation. IL-6 activates STAT3, which translocates to the nucleus and drives hepcidin transcription (Nemeth and Ganz, 2023; Gordeuk et al., 2011). TNF-α and interleukin-1 beta (IL-1β) synergistically potentiate this effect via NF-*κ*B-mediated chromatin remodeling, while also suppressing ferroportin transcription through BACH1-dependent mechanisms (Matsuoka et al., 2024). Excess hepcidin sequesters iron within macrophages and enterocytes, creating functional iron deficiency, adequate stores but reduced bioavailability, that limits heme synthesis, downregulates EPOR, and impairs JAK2/STAT5 signaling, collectively exacerbating EPO resistance (Komada et al., 2018). Pyroptotic cell death in the kidney further reduces the REPC pool, compounding EPO deficiency (Souma et al., 2016).

**FIGURE 4:**
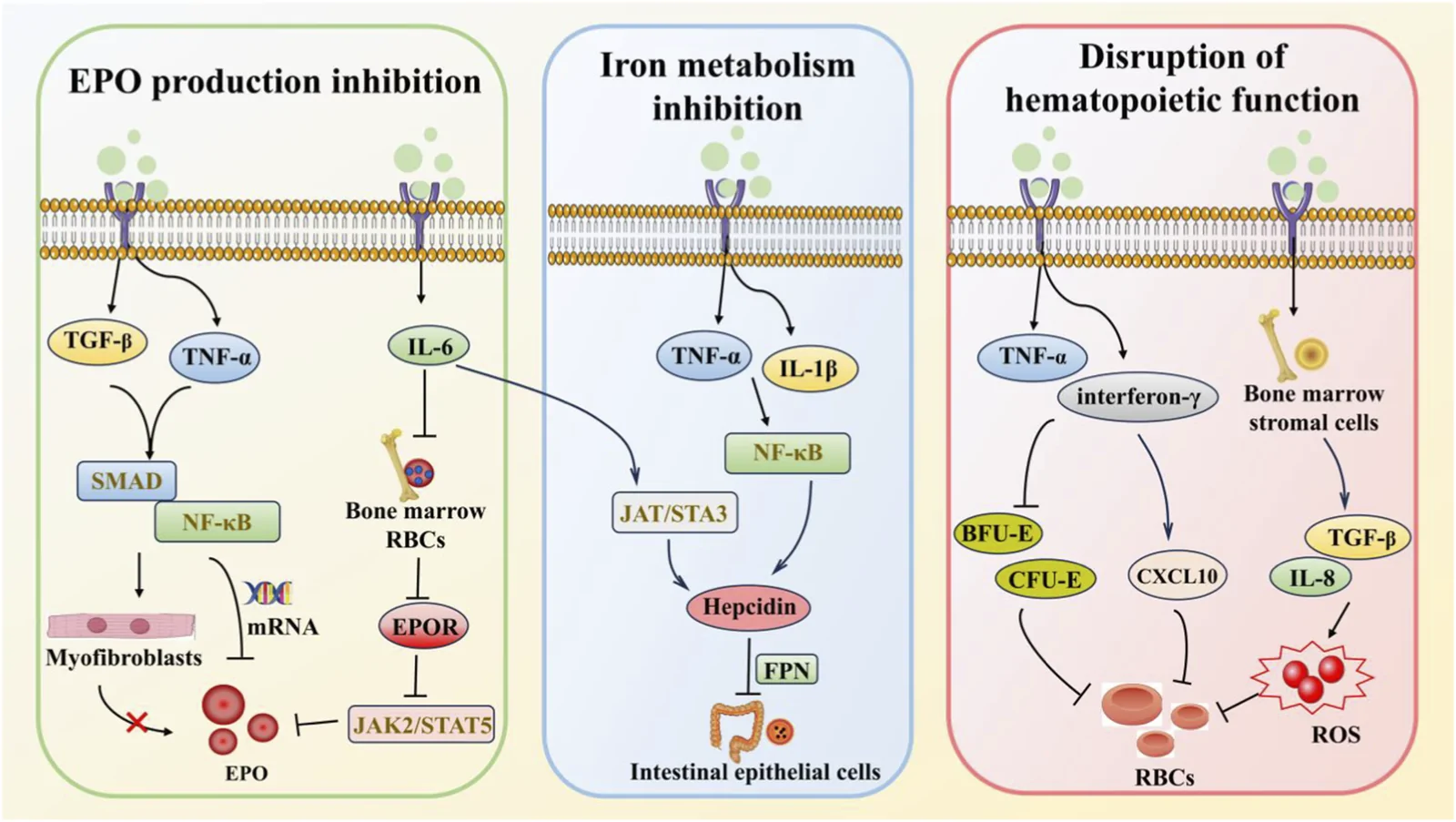
Inflammation-induced RA involves several pathological mechanisms.

Negative feedback regulators such as SOCS normally constrain inflammatory responses by attenuating cytokine receptor signaling (Kile and Alexander, 2001). In RA, however, chronic inflammation and impaired growth hormone signaling are associated with elevated SOCS expression, which may reduce erythroid progenitor responsiveness to EPO through modulation of JAK2/STAT5 signaling (Gluba-Brzózka et al., 2020). Thus, inflammation drives erythropoiesis suppression through interconnected pathways: impairing renal EPO production, inducing functional iron deficiency, and directly inhibiting bone marrow progenitor function (Figure 4), while initiating a vicious cycle through inflammasome activation and SOCS dysregulation that sustains persistent anemia and erythropoietic failure.

### 2.5 Other factors

In addition to EPO deficiency and iron dysregulation, RA is also shaped by a constellation of interrelated systemic factors, such as gut microbiota dysbiosis, mitochondrial dysfunction, oxidative stress, and epigenetic reprogramming, all of which converge on a common pathogenic nexus.

Gut microbiota dysbiosis contributes to RA pathogenesis. In CKD, urea accumulation promotes urease-producing bacterial overgrowth, disrupts the intestinal barrier, and facilitates endotoxin translocation into circulation, triggering systemic inflammation and hepcidin upregulation (Zheng et al., 2023). Elevated hepcidin sequesters iron in reticuloendothelial stores, causing functional iron deficiency. Meanwhile, gut microbial metabolism of amino acids generates uremic toxins such as indoxyl sulfate and p-cresyl sulfate that directly suppress erythropoiesis and reduce EPO responsiveness (Taguchi et al., 2019). Recent studies have shown that natural products and functional foods exert pharmacological effects by modulating the gut microbiota. Chang et al. systematically reviewed the influence of functional foods and dietary supplements on metabolic diseases via gut microbiota regulation, highlighting pathways related to inflammation and iron metabolism (Chang et al., 2025). Wang et al. further proposed that metabolic reprogramming of the gut microbiota drives the development of host metabolic disorders (Wang et al., 2026). These findings are relevant to TCM, as many oral TCM formulas and natural products may alleviate RA by restoring gut microbial balance, lowering uremic toxin production, and reducing inflammation-driven hepcidin expression.

The uremic environment, characterized by the retention of protein-bound toxins such as indoxyl sulfate and p-cresyl sulfate, directly inhibits RBCs. These toxins enter cells via organic anion transporters, activate the aryl hydrocarbon receptor signaling pathway, and disrupt mitochondrial iron-sulfur cluster assembly in erythroid progenitors, thereby inhibiting heme synthesis and promoting EPO resistance (Taguchi et al., 2019). Importantly, the production of these uremic toxins is heavily dependent on gut microbial metabolism, establishing a direct link between intestinal dysbiosis and the accumulation of erythropoiesis-suppressing compounds.

In parallel, phosphate accumulation stimulates osteoblasts to secrete fibroblast growth factor 23 (FGF23), which inhibits renal 1-α-hydroxylase, reduces calcitriol production, and stimulates PTH secretion. FGF23 directly suppresses erythroid progenitor proliferation and downregulates EPOR expression, whereas PTH induces bone marrow fibrosis, inhibits erythroid progenitors, and increases RBC membrane fragility (Zadrazil and Horak, 2015). These factors are not independent: uremic toxins stimulate FGF23 secretion, and FGF23-induced calcitriol suppression raises PTH levels, while PTH-driven bone resorption releases additional uremic toxins, forming a self-reinforcing cycle that progressively impairs the hematopoietic microenvironment.

Cell senescence serves as a unifying framework that integrates these diverse pathological inputs, characterized by cell cycle arrest and the senescence-associated secretory phenotype, which involves secretion of inflammatory factors and matrix remodeling enzymes (Kooman et al., 2017). Senescent cells continuously induce local inflammation and fibrosis in REPCs, exacerbating EPO deficiency, impairing hematopoietic support in the bone marrow microenvironment, and driving mitochondrial DNA damage while activating the cGAS-STING signaling pathway to amplify the senescence-associated secretory phenotype (Švajger et al., 2025; Lai et al., 2025). Thus, senescence is not only a consequence of cumulative uremia and endocrine stress but also a driving force behind progressive, self-perpetuating tissue dysfunction. Gut microbiota dysbiosis may accelerate this senescent cascade by fueling chronic inflammation and oxidative stress, thereby contributing to the vicious cycle of RA progression.

## 3 Conventional and TCM-based therapeutic strategies for RA

Current clinical management of RA primarily relies on recombinant human erythropoietin (rhEPO), hypoxia-inducible factor prolyl hydroxylase inhibitors (HIF-PHIs), iron supplementation, and RBC transfusion. However, the modalities are not without limitations: ESA hyporesponsiveness remains a frequent clinical challenge; the long-term cardiovascular safety of HIF-PHIs remains controversial; and iron overload from supplementation and transfusion poses additional risks. These unresolved challenges underscore the ongoing need for alternative or complementary therapeutic strategies, including TCM interventions, which may offer additional benefits in managing refractory anemia and mitigating treatment-related complications.

Given these unresolved challenges, a growing body of evidence has turned attention to TCM as a potential complementary strategy for RA management. Globally, herbal-based traditional and complementary medicine is widely used among end-stage renal disease patients on hemodialysis (Acquah-Mills et al., 2025). A retrospective clinical study employing propensity score matching demonstrated that the Yishenqinglihuoxue formula, when combined with conventional Western medicine, significantly improved renal function parameters (eGFR, BUN, and Cr), delayed dialysis initiation, and ameliorated anemia, systemic inflammation, and calcium-phosphorus metabolism in patients with stage 5 CKD (Wu et al., 2025). In animal studies, steamed Panax notoginseng was shown to alleviate RA in an adenine-induced CKD mouse model by promoting overall hematopoiesis, restoring renal EPO mRNA and bone marrow EPOR mRNA expression, and inhibiting the progression of renal injury (Gao et al., 2022). Systems biology approaches have further uncovered novel mechanisms underlying TCM action in kidney disease. The gut microbiota-immune axis, long non-coding RNAs, metabolite biomarkers, and DNA methylation have been identified as key pathways through which TCM interventions exert their therapeutic effects (Miao et al., 2022). Notably, the Ureic Clearance Granule has been shown to ameliorate CKD and renal fibrosis by reshaping microbial dysbiosis and modulating microbiota-derived bile acid metabolism (Liu et al., 2026), providing a novel gut-kidney axis perspective for TCM-based CKD management. Additionally, anthraquinones from Rheum officinale have demonstrated renal fibrosis-ameliorating effects (Miao and Vaziri, 2025), while hederagenin has shown therapeutic potential in glomerulonephritis and nephrotic syndrome through inhibition of the JAK/STAT pathway and regulation of ferroptosis signaling.

These findings have substantially expanded the mechanistic understanding of TCM in the context of RA and kidney disease (Zhang et al., 2019). A wide range of medicinal plants are commonly used to treat CKD and its associated complications, including RA. Representative herbs such as *Angelica sinensis* (Oliv.) Diels, *Astragalus membranaceus* (Fisch.) Bunge, *Rheum officinale* Baill., *Codonopsis pilosula* (Franch.) Nannf., *Ligusticum chuanxiong* Hort., and *Salvia miltiorrhiza* Bunge have demonstrated potential in improving renal function and indirectly alleviating anemia through anti-inflammatory and nephroprotective effects. These plants contain diverse bioactive compounds, including polysaccharides, saponins, anthraquinones, flavonoids, and alkaloids, that have been shown to inhibit renal tubular apoptosis, improve micro-inflammatory states, and regulate iron metabolism. Their therapeutic action extends beyond direct EPO supplementation, achieving anemia improvement through integrated mechanisms such as immune modulation, metabolic regulation, and enhancement of hematopoietic support.

Current evidence indicates that TCM-mediated regulation of EPO in RA operates through three core pathways. The first and most direct pathway involves stabilization of HIF-2α: active ingredients such as polysaccharides and flavonoids inhibit PHD activity, reduce ubiquitin-mediated degradation of HIF-2α, and consequently upregulate EPO transcription in renal interstitial fibroblasts and hepatocytes. This is exemplified by AT-1, a novel amide thiazole compound that directly binds to the HIF-2α-PAS-B domain with a Kd of 2.63 μM and enhances the stability of the HIF-2α-ARNT heterodimer, thereby promoting EPO transcription (Chu et al., 2026). The ethanol extract of *Gynura procumbens* stems has also been shown to significantly ameliorate adenine-induced alterations in HIF-2α and EPO expression in rat kidney tissues (Li T. T. et al., 2025). The second pathway involves inhibition of inflammatory signaling to relieve EPO suppression. Chronic microinflammation is a key mechanism underlying impaired EPO synthesis and EPO resistance in CKD. TCM components, including anthraquinones, saponins, and alkaloids, inhibit NF-*κ*B, JAK/STAT, and other inflammatory pathways, reduce the release of pro-inflammatory cytokines such as IL-6 and TNF-α, and thereby mitigate the inflammation-mediated suppression of EPO production and erythroid differentiation (Wang Y. D. et al., 2023). The baicalin-geniposide combination has been demonstrated to alleviate cerebral and renal inflammatory injury through modulation of the HIF-1α/EPO/NF-*κ*B pathway (Hu et al., 2025). The third pathway involves regulation of iron metabolism homeostasis to enhance EPO sensitivity. Hepcidin-driven iron dysregulation is a major cause of EPO hyporesponsiveness in CKD. TCM can downregulate hepcidin expression, promote iron mobilization and absorption, improve iron utilization efficiency, and thereby enhance the erythropoietic response to endogenous EPO. Clinical evidence from an RCT demonstrated that Majun-e-khabsul hadeed, a traditional Unani formulation containing ferric oxide and herbal components rich in vitamin C and flavonoids, significantly improved Hb and serum ferritin levels in women with iron deficiency anemia, with comparable efficacy and a superior safety profile compared to standard oral iron supplementation (Firdose et al., 2025). The integrative regulation of phosphatidylcholine metabolism through phospholipase A2 further illustrates how TCM interventions, such as Rheum officinale, restore metabolic homeostasis to support hematopoietic function in CKD (Wang Y. N. et al., 2023). HIF-2α stabilization, inflammation suppression, and iron metabolism regulation are three pathways that function in an integrated and mutually reinforcing manner, collectively constituting the mechanistic framework through which TCM interventions exert their anti-anemic effects. The following sections will elaborate on the active monomers and classic TCM formulations in relation to these three core EPO regulatory pathways. Key monomers, extracts, and traditional preparations investigated for RA treatment are summarized in Table 1.

**TABLE 1:** Traditional Chinese medicine for treating renal anemia.

Intervention	Route, dosage, and frequency	Research model	Mechanism	Result	Origin/form	References
Angelica sinensis polysaccharide	Gastric gavage, 0.5–1 g/kg, QD	Adenine-induced CKD anemic rats	Stabilize HIF-2α protein, anti-inflammatory effect, and regulation of iron metabolism	NF-*κ*B↓, GATA2↓, JAK2/STAT5↑, PI3K/Akt↑, Hb↑	Angelica Sinensis	Bi S. J. et al. (2021)
Astragalus polysaccharide	Gastric gavage, 200 mg/kg, QD	Iron dextran-induced mice	Anti-inflammatory, regulating iron metabolism	IL-6↓, NF-*κ*B↓, BMP/SMAD↓, ferroportin↑, Fe^2+^↑	Astragali Radix	Du et al. (2022)
Astragaloside IV	Oral, 5 g/kg/d	Streptozotocin-induced anemic mice	Reduce renal damage and interstitial fibrosis, and indirectly promote EPO regulation	MEK1/2-ERK1/2-RSK2↓, ROS↓, p62↑, Nrf↑	Astragali Radix	Li et al. (2017)
Rhein	Gastric gavage, 1 g/kg, QD	Adenine-induced CKD anemic rats	Anti-inflammatory effects and regulation of iron metabolism	NF-*κ*B↓, NLRP3↓, IL-1β↓, IL-6↓, TNF-α↓, hepcidin↓	Rhubarb	Ge et al. (2017)
Emodin	Gastric gavage, 5 mg/kg, QD	Renal ischemia-reperfusion in rats	Anti-inflammatory	TNF-α↓, IL-1β↓, IL-6↓; p38↓, p65↓, ERK↓, JNK↓	Rhubarb	Wang Y. Y. et al. (2022)
Codonopsis pilosula polysaccharide	Oral, 200 mg/kg/d	Cyclophosphamide-induced RA in BALB/c mice	Anti-inflammatory and iron-regulating pathways	NF-*κ*B↓, MAPK↓, IL-6↓, TNF-α↓, Fe^2+^↑	Codonopsis pilosula	Fu et al. (2018)
Luteolin	Gastric gavage, 100 mg/kg, QD	Hgcl_2_-induced anemia mice	Directly regulate the PHD2/HIF-2α pathway and upregulate the expression of the EPO gene; Anti-inflammatory	NF-*κ*B↓, IL-6↓, IL-8↓, IL-1β↓, TNF-α↓, HIF-2α↑	Resedaceae	Wang Q. et al. (2022)
Silkworm Feces extract	Gastric gavage, 54 mg/kg, QD	Adenine-induced CKD in rats	Inhibit the activity of PHD2 and directly regulate the expression of EPO	NF-*κ*B↓, GATA2↓, BMP6/SMAD4↓, IL-6/STAT3↓, HIF-2α↑	Silkworm	Huang et al. (2017)
Tetramethylpyrazine	Gastric gavage, 40 mg/kg, QD	Renal ischemia-reperfusion in rats	Anti-inflammatory and antioxidant properties	TNF-α↓, IL-6↓,NF-*κ*B↓, PPAR-γ↑	Ligusticum wallichii	Li and Gong (2022)
Tanshinone IIA	Gastric gavage, 10 mg/kg, QD	5/6 nephrectomy	Reduce kidney inflammation and interstitial fibrosis	TGF-β/Smad2/3↓, TNF-α↓, NF-*κ*B↓	Salvia miltiorrhiza	Huang et al. (2022)
Catalpol	Gastric gavage, 100 mg/kg, QD	AA-I induces RA mice	Anti-inflammatory, protecting renal function	IL-6↓, p-JAK2/p-STAT3↓, NF-*κ*B↓, Hepcidin↓, Nrf2↑	Rehmannia glutinosa	Gao et al. (2023)
Breviscapine	Oral, 40 mg/kg/d	Adenine-induced renal fibrosis in rats	Reduce inflammation and oxidative stress	TGF-β1/Smad↓, TGF-β1/ERK↓, Wnt/β-catenin↓	Erigeron breviscapus	Zhang et al. (2022)
Danggui Buxue decoction	Gastric gavage, 500 mg/kg, QD	Antibiotic-induced anemic mice	Regulate the HIF-2α/EPO axis, anti-inflammatory and antioxidant	NF-*κ*B↓, HIF-2α↑, Nrf2↑, Hb↑	Astragali Radix and Angelica sinensis	Wang et al. (2018)
Jianpi Yishen decoction	Gastric gavage, 1.36 g/kg, QD	5/6 nephrectomized rats	Upregulate the expression of HIF-1α, anti-inflammatory, regulate iron metabolism	JAK2-STAT3↓, NF-*κ*B↓, TGF-β/Smad↓, hepcidin↑, Hb↑	Astragali Radix, Rhizoma Atractylodis Macrocephalae, Chinese yam, Cistanche deserticola, Amomum villosum, Salvia miltiorrhiza, Rhubarb, Glycyrrhiza uralensis	Li, C. H. et al., 2023
Renshen Yangrong decoction	Oral, 100 mL, QD	Patients with RA	Anti-inflammatory, regulation of iron metabolism	IL-6↓, TNF-α↓, ferroportin↑, Fe^2+^↑, Hb↑	Ginseng, Astragali Radix, Rhizoma Atractylodis Macrocephalae, Poria cocos, Angelica sinensis, Paeoniae Radix Alba, Rehmanniae Radix Preparata, Tangerine peel, Cinnamomum cassia, Schisandra chinensis, Polygalae Radix and Glycyrrhiza uralensis	Sheng et al. (2019)
Siwu decoction	Gastric gavage, 0.2 mL/10 g, QD	Rats with blood deficiency syndrome	Promote the synthesis of EPO and have anti-inflammatory effects	TLR4/NF-*κ*B/NLRP3↓, Bcl-2↑, EPO↑	Rehmanniae Radix Preparata, Angelica sinensis, Paeoniae Radix Alba, and Chuanxiong	Wang et al. (2016)
Yixuesheng capsule	Gastric gavage, 0.39 g/kg, QD	Iron-deficiency anemic mice	Anti-inflammatory and iron-regulating pathways	TNF-α↓, VEGF-A↓, STAT3↓, Fe^2+^↑	Asini Corii Colla, Astragali Radix, Polygoni Multiflori Radix Praeparata	Xu et al. (2023)
Shengxuebao mixture	Oral, 5 mL, TID	Patients with RA	Anti-inflammatory	NF-*κ*B↓, TNF-α↓, Fe^2+^↑, Hb↑	Polygoni Multiflori Radix Praeparata, Ligustri Lucidi Fructus, Mori Fructus, Ecliptae Herba, Paeoniae Radix Alba, Astragali Radix, and Cibotii Rhizoma	Li, A. L. et al. (2023)
Shengxuening tablet	Oral, 0.5 g, TID	Patients with RA	Regulate iron metabolism	ferroportin↑, Fe^2+^↑, hepcidin↑, Hb↑	Silkworms and extract	Mei et al. (2021)
Fufang E’jiao Jiang	Gastric gavage, 12 mL/kg, QD	Adenine-induced CKD anemic rats	Promote EPO expression and anti-inflammatory effect	TGF-β↓, EPO↑, IL-1β↑, IL-3↑	Asini Corii Colla, Red Ginseng, Codonopsis pilosula, Rehmanniae Radix Preparata, and Hawthorn	Zhao et al. (2023)
Shen-Shuai-Ning capsule	Gastric gavage, 10 mL/kg, QD	Adenine-induced CKD in rats	Anti-inflammatory, protecting the kidneys	TNF-α↓, IL-1β↓, IL-6↓, NF-*κ*B↓	Pseudostellaria heterophylla, Coptis chinensis, Pinellia ternata, Tangerine Peel, Poria cocos, Rhubarb, Salvia miltiorrhiza, Achyranthes, Safflower, and Glycyrrhiza uralensis	Liu et al. (2014a)
Huangkui capsule	Gastric gavage, 4 mg/kg, QD	Rats with streptomycin-induced diabetic nephropathy	Anti-inflammatory, antioxidant and anti-fibrotic	BUN↓, TNF-α↓, IL-6↓, PPAR↑	Abelmoschus manihot	Yuan et al. (2023)

↑, increase; ↓, decrease; QD, quaque die; TID, ter in die.

### 3.1 General treatment of RA

#### 3.1.1 EPO stimulating agent

RA is a frequent complication of CKD, a condition fundamentally linked to deficient EPO production by renal peritubular fibroblasts. The severity of anemia correlates directly with the degree of endogenous EPO deficiency. Clinical studies have established that ESA therapy effectively elevates Hb levels, which may slow RA progression and reduce cardiovascular morbidity (Mimura et al., 2015). Since the FDA approval of recombinant human EPO in 1989, ESAs have remained the cornerstone of RA management. Sequential pharmacokinetic optimization has yielded three generations of agents: short-acting epoetin alfa/beta, which has a half-life of 6–8 h and is administered thrice weekly; darbepoetin alfa, with a half-life exceeding 25 h, allowing weekly to biweekly dosing; and pegylated EPO beta, which has a half-life of over 130 h, enabling monthly dosing (Malyszko and Malyszko, 2016). By activating the JAK2-STAT5 pathway in erythroid progenitors, these agents effectively increase Hb levels and reduce transfusion requirements, and exhibit comparable efficacy across various administration regimens (Macdougall, 2024; Schmid and Jelkmann, 2016). A systematic review by Alsalimy and Awaisu compared methoxy polyethylene glycol-epoetin beta with darbepoetin alfa in non-dialysis-dependent CKD patients and found that both agents effectively maintained Hb levels within the target range, with comparable efficacy and safety profiles (Alsalimy and Awaisu, 2014). A meta-analysis by Ou et al. (2026) evaluating different ESA regimens for RA included 28 studies comprising 1,952 patients. The analysis revealed no statistically significant differences in Hb increase between less frequent higher-dose epoetin regimens and more frequent lower-dose regimens, nor between darbepoetin and short-acting ESAs, confirming that various ESA administration strategies have comparable efficacy. Notably, the darbepoetin group exhibited a superior safety profile compared with short-acting ESA groups (Ou et al., 2026).

RhEPO and other ESAs effectively increase Hb, stimulate erythropoiesis, improve quality of life and survival in RA patients, and are widely used as alternative treatments for advanced renal disease (Zheng Q. Y. et al., 2020; Rainville et al., 2016). Despite their established efficacy, ESAs face two major challenges. The first is the cardiovascular risk of aggressive Hb correction; targeting Hb above 13 g/dL increases stroke, hypertension, and thromboembolic events. The second is ESA hyporesponsiveness, affecting 5%–30% of patients depending on definition, and correlates with higher mortality and cardiovascular risk (McCausland et al., 2025). This multifactorial condition arises from iron deficiency, chronic inflammation, hyperparathyroidism, malnutrition, and occasionally underlying malignancy (Mase et al., 2023). Current management prioritizes the systematic optimization of modifiable factors including iron stores, inflammation, and metabolic status, rather than indiscriminate dose escalation. For patients who remain refractory despite optimal care, HIF-PHIs offer a mechanistically distinct alternative by stimulating endogenous EPO production while suppressing hepcidin to improve iron utilization, thus circumventing the inflammatory barriers driving ESA resistance. This mechanistic advantage positions HIF-PHIs as a valuable option for ESA-refractory patients, and further highlights the potential of TCM, which may target analogous pathways including HIF-2α stabilization, inflammation modulation, and iron metabolism regulation, to serve as a complementary and integrative therapeutic strategy for this challenging patient population.

#### 3.1.2 Hypoxia-inducible factor prolyl hydroxylase inhibitors

Hypoxia-inducible factor prolyl hydroxylase inhibitors have been introduced as innovative therapeutic options for RA management (Nakanishi and Kuragano, 2024; Haase, 2017). HIF-PHIs promote endogenous EPO production by stabilizing HIF-α and suppress hepcidin to improve iron availability, thereby addressing both EPO deficiency and functional iron restriction in RA. This dual mechanism, which distinguishes HIF-PHIs from ESAs, enables effective Hb maintenance without dose escalation (Kuragano, 2024; Mima, 2021). The recognition of PHD as a key regulator of erythropoiesis has further expanded therapeutic strategies for RA (Li Z. L. et al., 2020).

The first wave of HIF-PHIs, such as roxadustat, has been approved for clinical use in many Asian and European countries. By maintaining physiological EPO levels and lowering hepcidin to improve iron availability, HIF-PHIs synergistically correct the dual pathologies of EPO deficiency and functional iron restriction. These drugs exhibit unique therapeutic advantages over conventional ESAs, maintaining target Hb concentration while avoiding the risk of ESA dose escalation (Tang et al., 2024; Liu J. et al., 2023). Extensive clinical evidence has confirmed that HIF-PHIs effectively treat RA. The systematic review and network meta-analysis by Ren et al., which included 47 studies and 55 randomized controlled trials (RCTs), showed that all six HIF-PHI drugs effectively increased Hb levels in CKD patients (Ren et al., 2024). Compared with ESA or placebo, the overall risk of adverse events did not increase, and no significant difference in safety was observed among the different drugs. Overall, HIF-PHIs are considered a safe and effective alternative for CKD anemia, although long-term cardiovascular safety remains an area of active investigation (Li J. et al., 2023). A 2024 meta-analysis published in *NEJM Evidence* (25 RCTs, 26,478 participants) showed no difference in cardiovascular safety compared with ESAs in dialysis-dependent CKD (RR 0.99; 95% CI 0.92–1.08), though the Italian Society of Nephrology cautions that current studies lack sufficient follow-up to rule out delayed adverse events such as cancer, death, or cardiovascular events (Ha et al., 2024; Locatelli et al., 2024). For ESA-hyporesponsive patients, HIF-PHIs offer a valuable alternative by suppressing hepcidin and improving iron utilization, thereby overcoming inflammatory barriers. Additionally, TCM interventions that target similar pathways may also serve as complementary options.

#### 3.1.3 Iron therapy

Iron metabolism dysregulation is a central feature of RA management, particularly in dialysis patients, where absolute or functional iron deficiency affects 80%–90% of individuals (Tanaka and Tanaka, 2015). The underlying mechanisms include inadequate intake, dialysis-related blood loss, and hepcidin-mediated iron sequestration. Intravenous iron surpasses oral iron due to poor gastrointestinal absorption and tolerability, effectively restoring iron stores while reducing ESA requirements by 30%–50%, thereby mitigating the risks associated with high-dose ESA therapy (Matsuoka et al., 2024). However, intravenous iron carries hypersensitivity risks, possibly linked to complement activation, and transfusion remains a rescue option despite its inherent risks of alloimmunization and iron overload (Borawski et al., 2021).

Substantial clinical evidence has established the efficacy of iron therapy for RA. A systematic review and meta-analysis by Salim et al., including four RCTs involving 632 hemodialysis patients, demonstrated that high-dose intravenous iron supplementation was superior to low-dose iron treatment in improving ferritin levels, transferrin saturation, and Hb, while also significantly reducing EPO requirements (Salim et al., 2019).

Novel oral iron agents, including ferric citrate and ferric maltol, have broadened therapeutic options for anemia in CKD patients (Womack et al., 2020). Ferric maltol, a stable complex of ferric iron and three maltol molecules, effectively elevates Hb, transferrin saturation, and serum ferritin levels, with favorable tolerability and minimal gastrointestinal toxicity attributed to its controlled iron release in the intestinal lumen (Pergola and Kopyt, 2021; Nah et al., 2020). Despite therapeutic advances, iron therapy necessitates careful monitoring to prevent iatrogenic overload, as non-transferrin-bound iron can drive Fenton-mediated oxidative stress, thereby increasing cardiovascular and infection risks. Current guidelines recommend maintaining transferrin saturation at 20%–40% and serum ferritin at 200–500 ng/mL to balance erythropoietic benefit against iron-related toxicity (Khalil et al., 2024). The paradigm shift from a management approach centered on ESAs to one centered on iron carries significant clinical implications. It also underscores the potential for TCM interventions that enhance iron absorption and utilization, for example, through gut microbiota modulation or hepcidin suppression, to serve as valuable complementary strategies.

#### 3.1.4 Transfusion therapy

Current therapeutic approaches prioritize ESAs and iron supplementation over transfusion for managing RA. In transplant candidates, transfusion should be avoided to prevent allosensitization and panel-reactive antibody formation, which prolong waiting times and increase rejection risk (Bohlius et al., 2019). In patients with malignancies, transfusion may be necessary for acute symptom control, whereas ESAs are primarily indicated for chemotherapy-related anemia and are not recommended for non-chemotherapy-related cases or those with curative potential (Bohlius et al., 2019). However, in certain high-risk populations, judicious transfusion may confer survival benefits. For instance, a large retrospective cohort study of 6,604 critically ill patients with sepsis and concurrent CKD from the MIMIC-IV database demonstrated that RBC transfusion was associated with a significantly lower risk of 28-day mortality after multivariate adjustment and propensity score matching, with the benefit being particularly pronounced in patients with a SOFA score ≥ 5, base excess < 3, or eGFR < 30 mL/min/1.73 m^2^ (Chen L. et al., 2024). These findings support the effectiveness of transfusion in select patients when alternative therapies are insufficient or contraindicated.

Despite these potential benefits, transfusion therapy must be administered with caution due to its inherent risks. Beyond allosensitization, these include transfusion-associated circulatory overload, particularly in patients with underlying cardiovascular disease, which may precipitate acute decompensated heart failure. Transfusion-related acute lung injury represents another serious complication, mediated by donor antibodies directed against recipient leukocytes (Kers and Florquin, 2015). Each unit of packed RBCs contains approximately 200–250 mg of iron, and repeated transfusions can rapidly lead to parenchymal iron deposition, with subsequent organ dysfunction affecting the myocardium, liver, and endocrine organs. Despite advances in infectious disease screening, transfusion still carries residual risks of pathogen transmission, including emerging infectious agents for which screening assays may not yet be available (Marsella and Borgna-Pignatti, 2014). Given these risks, transfusion decisions in RA require individualized risk-benefit assessment, accounting for transplant candidacy, cardiovascular status, and response to alternative therapies. When transfusion is indicated, the use of leukoreduced products, appropriate volumes, and HLA antibody monitoring in transplant candidates can help mitigate alloimmunization, circulatory overload, and other complications.

### 3.2 Monomers and extracts for the treatment of RA

#### 3.2.1 Angelica sinensis polysaccharide

Angelica sinensis is the dried root of *Angelica sinensis* (Oliv.) Diels, from the Umbelliferae family. Its traditional applications include invigorating blood, regulating menstruation, and relieving pain, while modern research has also revealed anti-inflammatory, anti-tumor, and immunomodulatory properties (Wei et al., 2016). Pharmacological studies have attributed these broad effects primarily to its active constituents, among which Angelica sinensis polysaccharide (ASP) is one of the core bioactive compounds. ASP is the principal water-soluble polysaccharide of Angelica sinensis and serves as a representative TCM polysaccharide with diverse biological activities. It is composed of multiple monosaccharides, including glucose, galactose, and xylose arabinose, with its acidic polysaccharide fraction also containing glucuronic acid and hemiglycuronic acid. These structural features underpin a wide spectrum of pharmacological effects, making ASP valuable in both TCM and modern medicine (Bi S. J. et al., 2021). Reported activities include hematopoiesis promotion, immunoenhancement, antitumor effects, and antioxidant properties. As the main bioactive component of Angelica sinensis used in the treatment of anemia, ASP has demonstrated significant therapeutic effects on iron deficiency anemia, anemia of chronic disease, and RA.

Preclinical studies have demonstrated that ASP exerts its hematopoietic effects through a multi-pathway synergistic mechanism that integrates HIF-2α stabilization, inflammation suppression, and iron metabolism regulation. In adenine-induced CKD anemia rat models, oral administration of ASP significantly elevates serum EPO concentration by stabilizing HIF-2α protein in renal and hepatic tissues to promote EPO transcription. Simultaneously, it downregulates NF-*κ*B signaling and GATA2 expression to suppress pro-inflammatory cytokine release, thereby relieving inflammatory inhibition of EPO synthesis. Additionally, ASP activates the JAK2/STAT5 and PI3K/Akt pathways downstream of the EPO receptor, upregulates anti-apoptotic proteins B-cell lymphoma-extra large (Bcl-xL) and Fam132b, increases the Bcl-2/Bax ratio in bone marrow mononuclear cells, inhibits erythroid progenitor apoptosis, and enhances the responsiveness of bone marrow hematopoietic cells to EPO (Wang et al., 2018). With respect to iron metabolism, ASP significantly inhibits hepcidin expression, restores iron homeostasis, and increases systemic iron availability, which in turn cooperates with elevated EPO to promote erythropoiesis (Wang et al., 2017). Furthermore, ASP delays hematopoietic stem cell senescence and promotes their proliferation, thereby exerting a multidimensional regulatory effect on hematopoiesis, although these findings await clinical confirmation.

#### 3.2.2 Astragalus polysaccharide

Astragalus membranaceus is the dried root of the legume *Astragalus membranaceus* (Fisch.) Bge. var. *mongholicus* (Bge.) Hsiao or *Astragalus membranaceus* (Fisch.) Bge. (Zhang et al., 2014). Modern pharmacological studies have demonstrated that Astragalus exhibits a broad spectrum of therapeutic effects, including anti-tumor activity, cardiovascular and cerebrovascular protection, immunomodulation, respiratory support, metabolic tissue protection, blood pressure regulation, anti-aging properties, and prevention of osteoporosis (Zheng Y. J. et al., 2020). These broad pharmacological effects are largely attributed to the rich chemical composition of Astragalus, which includes polysaccharides, saponins, flavonoids, amino acids, and alkaloids, among which Astragalus polysaccharide (APS) is one of the core bioactive constituents. APS consists of water-soluble and water-insoluble dextrans, as well as water-soluble acidic heteropolysaccharides, all of which exhibit multiple biological activities (Tian et al., 2025). As an immunomodulator, APS significantly enhances immune function and exerts anti-aging, anti-radiation, anti-stress, antioxidant, antiviral, anti-tumor, and hypoglycemic effects (Li C. X. et al., 2022; Du et al., 2022).

APS improves RA mainly through the inflammatory inhibition and iron metabolism regulation pathways (Ren et al., 2016). By inhibiting the NF-*κ*B signaling pathway, APS attenuates the suppressive effects of inflammatory cytokines such as TNF-α and IL-6 on erythropoiesis, thereby effectively improving anemia (Wang et al., 2017). In addition, APS modulates iron metabolism-related pathways by inhibiting IL-6/STAT3 and BMP/SMAD signaling, reducing hepcidin overexpression, and alleviating iron deficiency and utilization disorders (Varga et al., 2021). Furthermore, APS promotes iron mobilization from the liver and spleen by upregulating ferroportin expression, increasing serum iron levels, and stimulating EPO elevation, thereby further correcting anemia. Modern pharmacological studies have confirmed that APS acts synergistically with saponins to increase Hb and RBC counts, reflecting the multi-component and multi-target characteristics of TCM in regulating EPO and improving anemia (Jia et al., 2019).

#### 3.2.3 Astragaloside IV

Astragaloside IV (AS-IV) is the primary saponin component of Astragali Radix in commonly used TCM. It exhibits a broad spectrum of pharmacological activities, including antioxidant, cardioprotective, anti-inflammatory, antiviral, antibacterial, anti-fibrotic, anti-diabetic, and immunomodulatory effects. Notably, in the context of kidney disease, AS-IV can reverse renal injury or delay disease progression through multiple pathways and targets (Li et al., 2017).

In the context of RA, AS-IV exerts indirect EPO-regulatory effects primarily by alleviating renal injury and interstitial fibrosis. AS-IV inhibits the MEK1/2-ERK1/2-RSK2 signaling pathway (Song et al., 2018). It also enhances p62 phosphorylation via the p62/Keap1/Nrf2 pathway, thereby promoting Nrf2 nuclear translocation, reducing ROS accumulation and renal fibrosis, preserving the number and function of renal interstitial EPO-producing cells, and restoring endogenous EPO secretion (Gao et al., 2021). In addition, AS-IV suppresses TGF-β/Smad and PI3K/Akt/mTOR signaling, delays renal tubular epithelial-mesenchymal transition, and exerts multi-target renoprotective effects, which provide a structural basis for the recovery of EPO synthesis (Lian et al., 2022).

#### 3.2.4 Rhein

Rhubarb is derived from the dried roots and rhizomes of *Rheum palmatum* L., *Rheum tanguticum Maxim*. ex Balf., and *Rheum officinale* Baill. Studies on the bioactive constituents of rhubarb mainly focus on anthraquinones, dianthrones, tannins, and polysaccharides (Xiang et al., 2020). Rhein, an anthraquinone compound with the chemical name 1,8-dihydroxy-3-carboxyanthraquinone, exhibits multiple biological activities, including antitumor, antibacterial, anti-inflammatory, anti-diabetic nephropathy, anti-hepatic fibrosis, and antiviral effects (Zhou et al., 2015).

Preclinical studies have shown that rhein improves RA through the dual pathways of anti-inflammation and iron metabolism regulation. It significantly reduces the production of pro-inflammatory cytokines such as IL-6, IL-1β, and TNF-α by inhibiting NF-*κ*B signaling and NLRP3 inflammasome activation, thereby relieving inflammatory suppression of EPO transcription and counteracting EPO resistance (Zhang et al., 2023; Ge et al., 2017). Meanwhile, rhein downregulates hepcidin expression, rectifies iron dysregulation, increases iron availability for erythropoiesis, and enhances the erythropoietic response to EPO (Yu et al., 2015). In terms of renal protection, rhein prevents renal interstitial fibrosis, preserves tubular structure, and maintains EPO secretion by renal interstitial cells, consistent with the effects of rhubarb extract in improving phosphatidylcholine metabolism and delaying CKD progression in adenine-induced models. However, direct evidence of EPO modulation by rhein has not been established, and its proposed EPO-regulatory effects remain to be confirmed through studies specifically designed to measure EPO expression and activity.

#### 3.2.5 Emodin

Emodin, a major bioactive anthraquinone derivative derived from rhubarb, exhibits a broad spectrum of pharmacological activities, including anti-inflammatory, immunomodulatory, anti-fibrotic, anti-tumor, antiviral, antibacterial, and anti-diabetic effects, along with significant renoprotective properties (Stompor-Gorący, 2021).

Emodin exerts anti-inflammatory effects by regulating the NF-*κ*B and mitogen-activated protein kinase (MAPK) signaling pathways, significantly inhibiting the expression of pro-inflammatory cytokines such as TNF-α, IL-1β, and IL-6, and reducing phosphorylation of p38, p65, extracellular signal-regulated kinase (ERK) and c-Jun N-terminal kinase (JNK). In ischemia-reperfusion renal injury models, emodin inhibits protein kinase 2 activation, blocks the pro-inflammatory effects of NF-*κ*B and MAPK pathways, and attenuates renal tubular epithelial cell injury, thereby preserving endogenous EPO secretion (Wang Y. Y. et al., 2022; Zhang et al., 2023). Furthermore, emodin, together with other rhubarb anthraquinones, increases glomerular filtration rate, corrects acid-base and electrolyte disturbances, exerts anti-oxidative stress effects, delays the decline of residual renal function, and indirectly ameliorates EPO synthesis impairment caused by progressive renal injury (Zheng et al., 2021).

#### 3.2.6 Codonopsis pilosula polysaccharide

The botanical sources of Codonopsis Radix include the dried roots of *Codonopsis pilosula* (Franch.) Nannf., *Codonopsis pilosula* var. *modesta* (Nannf.) L. T. Shen, and *Codonopsis tangshen* Oliv., all of which belong to the family Campanulaceae (Liang et al., 2024). Codonopsis pilosula s traditionally used to enhance systemic energy metabolism, support gastrointestinal and pulmonary function, promote hematopoiesis, and improve general constitutional health. Modern research has revealed that Codonopsis pilosula contains diverse bioactive compounds, including alkaloids, terpenes, alkynes, flavonoids, lignins, steroids, and saccharides, which are responsible for its broad spectrum of pharmacological activities (Ross, 2003). Clinically, it is indicated for symptoms related to spleen and lung deficiency, including poor appetite, fatigue, weak cough, and anemia-related manifestations such as sallow complexion and palpitations, as well as dyspnea, dry mouth, and thirst with a sensation of internal heat (Shi et al., 2024). Among its active constituents, Codonopsis pilosula polysaccharide (CPP) is a key extract that exhibits significant immunomodulatory and hematopoietic effects (Luan et al., 2021).

CPP improves RA primarily through anti-inflammatory and iron-regulating pathways. It inhibits NF-*κ*B and MAPK signaling, reduces the production of pro-inflammatory cytokines such as IL-6 and TNF-α, and attenuates the inflammatory suppression of erythropoiesis and EPO synthesis (Fu et al., 2018). By relieving chronic inflammation, CPP downregulates hepcidin expression induced by inflammatory signals, improves iron utilization, and enhances hematopoietic responsiveness to EPO. Furthermore, based on both traditional use and modern pharmacological evidence, Gao et al. (2018) found that Codonopsis pilosula significantly enhances hematopoietic function with low toxicity and minimal side effects, supporting its applicability as both a medicinal and dietary product.

#### 3.2.7 Luteolin

Luteolin (Lut) is a naturally occurring flavonoid, originally isolated from the stems, leaves, and branches of certain plants within the genus Reseda and other families (Hu et al., 2022). As a polyhydroxyflavonoid, Lut is widely distributed in Brassicaceae, Araceae, Apiaceae, Verbenaceae, and other plant families. It is currently obtained primarily through plant extraction. Despite its poor water solubility, Lut exhibits a broad spectrum of significant biological activities, including anti-inflammatory, antibacterial, antioxidant, and anti-tumor effects (Wang Q. et al., 2022).

Unlike the aforementioned components that act primarily indirectly, luteolin directly targets the PHD2/HIF-2α pathway to upregulate EPO expression while also exerting anti-inflammatory effects. In HgCl_2_-induced RA mouse models, luteolin acts as a PHD2 inhibitor to upregulate HIF-2α and EPO protein levels, directly promoting EPO transcription and synthesis, while partially alleviating oxidative stress injury to restore renal EPO secretion (Li S. Y. et al., 2020). Further studies in renal fibrosis-associated anemia models confirmed that luteolin significantly improved anemia parameters and renal function, upregulated EPO and HIF-2α expression, and downregulated α-smooth muscle actin, collagen I, and fibronectin levels. Mechanistically, luteolin improves renal fibrosis-related anemia by activating the SIRT1/FOXO3 pathway, while concurrently inhibiting NF-*κ*B signaling to reduce pro-inflammatory cytokines such as IL-6, IL-8, and IL-1β, thereby further potentiating the EPO-mediated erythropoietic effect (Li F. et al., 2022; Mahdiani et al., 2022).

#### 3.2.8 Silkworm feces extract

Silkworm dung, the dried feces of *Bombyx mori* (Linnaeus), has a long history of use in TCM for treating blood deficiency and rheumatic pain, with its anemia-related applications recorded as early as the Song Dynasty in *Neijing Shiyi Fanglun* and later in the Ming Dynasty *Bencao Gangmu*. The active extract, silkworm fecal extract (SFE), is a water-soluble iron complex primarily composed of sodium iron chlorophyllin and chlorophyll derivatives. SFE is prepared through a sequential process: chlorophyll is first extracted from silkworm dung, followed by removal of magnesium ions from the porphyrin ring to yield chlorophyll derivatives, which are then complexed with iron to form sodium iron chlorophyllin. Sodium iron chlorophyllin not only directly participates in Hb synthesis but is also efficiently absorbed via intestinal mucosal heme receptors. Furthermore, as a divalent, soluble, organic, and stable iron formulation, SFE enhances intestinal iron absorption (Huang et al., 2017).

In regulating EPO directly, SFE upregulates HIF-2α expression by inhibiting PHD2 activity, reduces HIF-2α protein degradation, and consequently promotes EPO transcription and synthesis in renal and hepatic tissues. Concurrently, it suppresses NF-*κ*B and GATA2 signaling, alleviating inflammatory inhibition of EPO and further enhancing its expression (Mei et al., 2021). Regarding iron metabolism, SFE significantly inhibits hepcidin expression by blocking BMP6/SMAD4 and IL-6/STAT3 signaling, restores iron homeostasis, increases bioavailable iron, and enhances the erythropoietic efficiency of EPO (Zeng et al., 2020).

#### 3.2.9 Tetramethylpyrazine

Ligusticum wallichii (Chuan Xiong), a plant in the Apiaceae family, has been documented in the *Shennong Bencao Jing* for its medicinal use of dried roots. Tetramethylpyrazine (TMP), an important alkaloid and one of its core bioactive compounds (Zhao et al., 2016), was first isolated in 1970 and has since demonstrated significant therapeutic potential across various diseases. TMP exerts a wide range of pharmacological effects, including cardiovascular actions such as vasodilation, microcirculation improvement, and antiplatelet aggregation, as well as organ-specific protection through renal vasodilation and cerebrovascular safeguarding. These properties underpin its broad clinical application in cerebrovascular disease, pulmonary hypertension, chronic renal failure, liver cirrhosis, and radiation-induced pulmonary fibrosis (Qi et al., 2024). TMP significantly attenuates tissue damage and promotes functional recovery via improved blood circulation and anti-oxidative stress. For example, it enhances cerebral perfusion by vasodilation and platelet inhibition in cerebrovascular disorders, while in chronic renal failure, it slows functional decline through renal vasodilation and anti-inflammatory mechanisms (Lin et al., 2022).

TMP improves RA primarily through anti-inflammatory and antioxidant pathways. It inhibits NF-*κ*B signaling by targeting TLR4 and TNFR or activating peroxisome proliferator-activated receptor-gamma (PPAR-γ), reduces pro-inflammatory cytokine release, and alleviates renal interstitial inflammation-mediated suppression of EPO synthesis (Li and Gong, 2022). In renal ischemia-reperfusion models, TMP downregulates NLRP3 inflammasome expression, reduces renal TNF-α and IL-6 levels, and attenuates tubular injury, thereby protecting the function of renal interstitial EPO-producing cells (Sun et al., 2020). In addition, TMP dilates renal vessels, improves renal microcirculatory perfusion, enhances renal tissue oxygen supply, and optimizes the microenvironment for EPO production by renal interstitial fibroblasts, thereby facilitating the restoration of endogenous EPO secretion (Gong et al., 2019).

#### 3.2.10 TanshinoneIIA

*Salvia miltiorrhiza* Bge. (Lamiaceae), first recorded in the *Shennong Bencao Jing* and used medicinally in China for millennia, is valued for its properties in activating blood circulation, resolving stasis, relieving pain, cooling blood, and calming the mind (Cai et al., 2024; Bi Z. Y. et al., 2021). Tanshinone IIA (Tan IIA), the most abundant lipophilic diterpenoid quinone and a core bioactive constituent of this herb, has been extensively studied as a representative compound. Tan IIA exhibits multifaceted biological activities, including antioxidant, anti-inflammatory, anti-fibrotic, antiviral, antitumor, antiplatelet, neuroprotective, and hepatoprotective effects, underpinning its therapeutic potential against diverse pathological conditions (Huang et al., 2022).

Preclinical studies have demonstrated that Tan IIA exerts anti-fibrotic and anti-inflammatory effects in kidney disease models, suggesting a potential indirect benefit for EPO production. In CKD rat models, Tan IIA significantly reduced urinary protein excretion and serum creatinine, alleviated glomerulosclerosis and renal inflammation, and may contribute to the preservation of renal endogenous EPO synthesis (Wang et al., 2015). Further mechanistic studies revealed that Tan IIA inhibited overactivation of the TGF-β/Smad2/3 signaling pathway and downregulated renal expression of fibronectin and collagens type III and IV (Bi Z. Y. et al., 2021). Meanwhile, Tan IIA suppresses NF-*κ*B signaling, reduces the production of TNF-α, monocyte chemoattractant protein-1, and other chemokines, and alleviates inflammatory damage to renal EPO-producing cells (Chen et al., 2023). The proposed EPO-modulatory effects of Tan IIA, however, are inferred from its renoprotective actions and await direct experimental validation.

#### 3.2.11 Catalpol

*Rehmannia glutinosa* Libosch. is a plant of the family Scrophulaceae. As one of the famous “four medicinal herbs,” its medicinal history can be traced back to *Shennong Bencao Jing*. The dried root of Rehmannia glutinosa is its main medicinal part, which is widely cultivated at home and abroad (Fu et al., 2023). Catalpol (CAT) is an iridoid glycoside compound isolated from the root. It has a variety of significant biological activities, including anti-inflammatory, anti-tumor, neuroprotective, and hypoglycemic effects. In recent years, CAT has gained particular attention for its renoprotective properties, especially in ameliorating RA and renal fibrosis (Gao et al., 2023).

Rehmannia glutinosa has been shown to protect renal function, support kidney health, and exert diuretic effects, with its active component CAT demonstrating significant efficacy in improving RA. In a mouse model of RA induced by aristolochic acid I (AA-I), catalpol significantly suppressed IL-6/p-JAK2/p-STAT3 signaling, reduced hepcidin levels, and corrected iron dysregulation, thereby enhancing EPO sensitivity (Liu Z. H. et al., 2024). Concurrently, catalpol restored the balance between Nrf2 and NF-*κ*B by upregulating Nrf2 and downregulating NF-*κ*B, activated antioxidant gene expression, reduced inflammatory cytokines, inhibited apoptosis, and attenuated the inflammation- and oxidative stress-mediated suppression of EPO synthesis (Liu J. Y. et al., 2023).

#### 3.2.12 Breviscapine

Erigeron breviscapus, also known as Dong Ju in Chinese, is a medicinal plant whose dried whole herb is derived from *Erigeron breviscapus* (Vant.) Hand.-Mazz. Breviscapine, the flavonoid extract from Erigeron breviscapus, is the effective constituent, mainly containing scutellarin along with a small amount of apigenin-7-O-glucuronide (Wen et al., 2021). Previous studies have suggested that breviscapine may attenuate renal fibrosis in patients with diabetic nephropathy, and this effect is associated with improved renal function, as evidenced by reduced proteinuria and lower serum creatinine levels (Su et al., 2024).

Breviscapine has demonstrated significant efficacy in treating kidney diseases, primarily through its phenolic and flavonoid constituents, which exert protective effects by reducing inflammatory cytokines, enhancing antioxidant enzyme activity, and mitigating damage to the hematopoietic microenvironment and RBCs induced by inflammation and oxidative stress (Wen et al., 2021). In renal fibrosis rat models, breviscapine inhibits TGF-β1/Smad and TGF-β1/ERK signaling, delays renal interstitial fibrosis progression, and preserves the EPO-secreting function of renal interstitial cells. It also attenuates inflammation- and oxidative stress-mediated injury to the bone marrow hematopoietic microenvironment and RBCs, thereby enhancing the erythropoietic efficacy of EPO (Zhang et al., 2022). Furthermore, breviscapine dilates blood vessels, reduces blood viscosity, and improves renal and bone marrow microcirculation, providing a favorable microenvironment for EPO-driven hematopoiesis. Its inhibitory effect on the Wnt/β-catenin pathway also contributes to renal protection, indirectly supporting the recovery of EPO synthesis (Huang B. R. et al., 2024).

### 3.3 TCM preparation for the treatment of RA

#### 3.3.1 Danggui Buxue decoction

Danggui Buxue Decoction (DBD), first recorded in the *Neiwaishang Bianhuo* Lun by Li Dongyuan during the Jin-Yuan Dynasties, comprises Astragali Radix and Angelicae Sinensis Radix. It is clinically indicated for anemia, fever due to metabolic imbalance, general debility, fatigue, internal injuries, menstrual disorders, and chronic ulcers (Sun L. et al., 2022). Modern pharmacological studies have demonstrated that DBD exerts multiple effects, including immunomodulation, hematopoietic promotion, and cardiocerebrovascular protection. Astragali Radix, the sovereign herb, enhances systemic energy metabolism, boosts immune function, and improves RA symptoms by promoting blood production. Mechanistically, Astragali Radix stimulates erythroid progenitor proliferation and differentiation, enhances erythropoiesis, and improves microcirculation and oxygen supply, thereby facilitating Hb synthesis (Ren et al., 2016; Li C. X. et al., 2022). As the ministerial herb, Angelicae Sinensis Radix upregulates renal and hepatic EPO expression, regulates key iron metabolism proteins such as hepcidin, promotes iron mobilization, and improves hemorheological parameters (Ma et al., 2015; Wang C. C. et al., 2016; Yang et al., 2014).

The combination of Astragali Radix and Angelicae Sinensis Radix forms a synergistic system wherein the former invigorates vital energy to enhance systemic metabolism and transport, thereby providing a substrate for the latter to promote hematopoiesis. In turn, Angelicae Sinensis Radix activates blood circulation and improves microcirculation, facilitating the distribution and absorption of active compounds from Astragali Radix. This synergistic interplay achieves multitarget amelioration of anemia by modulating the HIF-2α/EPO axis, the NF-*κ*B inflammatory pathway, and the Nrf2 antioxidant pathway (Wang et al., 2018; Yang et al., 2021). Metabolomics and systems pharmacology studies have shown that DBD indirectly improves renal function and alleviates RA by acting on multiple targets, including ABCG2, UGT1A8, and CYP3A4, and by modulating biotin synthesis and bile acid secretion pathways (Tie et al., 2022). A systematic review and meta-analysis of seven RCTs involving 460 patients found that adding DBD to conventional Western medicine for RA significantly improved RBC count and hematocrit, as well as overall clinical efficacy, although the Hb benefit was significant only when the decoction was prepared at the classical 5:1 ratio of Radix Astragali to Angelicae Sinensis (Zhao et al., 2017). Recent studies have further indicated that DBD may enhance its therapeutic effects by regulating the gut microbiota and improving the bioavailability of its active constituents (Chen et al., 2021).

#### 3.3.2 Jianpi Yishen decoction

Jianpi Yishen (JPYS) decoction is a TCM decoction composed of eight herbs, including Astragali Radix, Rhizoma Atractylodis Macrocephalae, Chinese yam, Cistanche deserticola, Amomum villosum, Salvia miltiorrhiza, Rhubarb and Glycyrrhiza uralensis. This prescription is indicated for strengthening the spleen and tonifying the kidney, while also eliminating turbidity and relieving stasis. Mechanistically, JPYS decoction enhances immune and hematopoietic functions through Astragali Radix and Cistanche deserticola, thereby promoting erythropoiesis (Liu et al., 2021). Rhizoma Atractylodis Macrocephalae and Chinese yam regulate gastrointestinal absorption and glycolipid metabolism, alleviating malnutrition and renal oxidative damage. Amomum villosum and rhubarb exert anti-inflammatory and detoxifying effects, reducing renal burden. Salvia miltiorrhiza improves microcirculation, inhibits fibrosis, and protects renal function, while Glycyrrhiza uralensis harmonizes the formulation and mitigates gastrointestinal irritation. Collectively, this multi-herb formula synergistically ameliorates anemia-related micro-inflammation, iron dysregulation, and toxin accumulation, while addressing both spleen-kidney deficiency and turbid stasis (Liu et al., 2018).

JPYS decoction regulates EPO through a multi-pathway synergistic mechanism: it upregulates HIF-1α expression in renal tissue to directly promote EPO transcription and synthesis; inhibits NF-*κ*B and TGF-β/Smad signaling to exert anti-inflammatory and anti-fibrotic effects, thereby preserving renal EPO-secreting function; and suppresses JAK2-STAT3 signaling to reduce hepcidin levels, regulate iron metabolism, and improve EPO responsiveness (Chen J. P. et al., 2018; Li C. H. et al., 2023). In adenine-induced nephropathy anemia rat models, JPYS alleviates RA symptoms by inhibiting JAK2-STAT3 activation, lowering hepcidin, and correcting iron dysregulation. Its beneficial effects also extend to anti-fibrotic and anti-inflammatory actions through suppression of NF-κB and TGF-β/Smad pathways, thereby restoring EPO synthesis. In 5/6 nephrectomized CKD rat models, JPYS upregulated EPO expression and inhibited renal fibrosis, further confirming its renoprotective and anti-anemic effects (Chen et al., 2019; Huang B. R. et al., 2024).

#### 3.3.3 Renshen Yangrong decoction

Renshen Yangrong decoction (RYD), derived from the *Taiping Huimin Heji Jufang*, is composed of Ginseng Radix et Rhizoma, Astragali Radix, Rhizoma Atractylodis Macrocephalae, Poria cocos, Angelicae Sinensis Radix, Paeoniae Radix Alba, Rehmanniae Radix Preparata, Citri Reticulatae Pericarpium, Cinnamomi Ramulus, Schisandrae Chinensis Fructus, Polygalae Radix, and Glycyrrhizae Radix et Rhizoma (Sheng et al., 2019). In this formulation, Ginseng Radix et Rhizoma enhances systemic metabolism, maintains fluid homeostasis, and supports hematopoiesis, acting synergistically with Astragali Radix, Rhizoma Atractylodis Macrocephalae, Poria cocos, and Glycyrrhizae Radix et Rhizoma to improve digestive and absorptive capacity. Citri Reticulatae Pericarpium regulates gastrointestinal motility and facilitates nutrient assimilation for blood and energy production. Rehmanniae Radix Preparata promotes erythropoiesis, renal function, and bone marrow activity, working in concert with Angelicae Sinensis Radix and Paeoniae Radix Alba to optimize hematopoietic outcomes. Schisandrae Chinensis Fructus supports renal metabolic function, while Polygalae Radix coordinates cardiovascular and neurological activity, promoting mental calmness and nervous system stability. Modern pharmacological studies have demonstrated that RYD contains various bioactive compounds, including saponins, carbohydrates, amino acids, and vitamins, which significantly enhance immune function and improve disease resistance (Deng, 2014).

RYD improves RA through dual regulation of inflammation and iron metabolism. It inhibits pathogenic autoreactive T-cell differentiation, reduces pro-inflammatory cytokines IL-6 and TNF-α, alleviates chronic inflammation, and relieves inflammatory suppression of EPO synthesis and erythropoiesis (Hsiao et al., 2015). Clinical studies have shown that RYD exerts significant therapeutic effects in maintenance hemodialysis patients with RA, with mechanisms involving reduced hepcidin levels, increased serum iron and ferritin, and improved iron utilization, thereby effectively ameliorating anemia. In addition, RYD modulates fibrin degradation product levels, further enhancing its therapeutic efficacy (Sun, 2023).

#### 3.3.4 Siwu decoction

Siwu decoction (SWD), as a classic prescription in TCM, is included in the *Taiping Huimin Heji Jufang*, which was compiled during the Song Dynasty. This prescription is composed of four medicinal herbs: Rehmanniae Radix Preparata, Angelica Sinensis, Paeoniae Radix Alba, and Chuanxiong. Within this herbal combination, Rehmanniae Radix Preparata promotes erythropoiesis, provides essential nutrients, and supports bone marrow activity. Angelica sinensis enhances blood production and improves microcirculation. Paeoniae Radix Alba contributes to hematopoietic function while modulating hepatic metabolism. Chuanxiong facilitates blood flow and optimizes tissue perfusion (Wang R. et al., 2016). This prescription is designed to replenish blood without causing stasis and to nourish without greasiness, addressing both blood deficiency and circulatory stagnation. Historically regarded by TCM practitioners as a fundamental blood-regulating prescription, SWD has served as an important reference for the treatment of blood deficiency syndromes in subsequent generations (Fang M. et al., 2024).

As a classic prescription of TCM, SWD is primarily indicated for nutrient blood deficiency with circulatory stasis, and its traditional therapeutic action is to activate blood flow and enhance peripheral circulation. SWD regulates EPO and hematopoiesis through multiple interconnected pathways. It upregulates renal EPO mRNA expression and promotes EPO synthesis and secretion. It also modulates the bone marrow hematopoietic microenvironment by upregulating Bcl-2 expression, inhibiting bone marrow cell apoptosis, and enhancing hematopoietic cell responsiveness to EPO. Furthermore, SWD suppresses the TLR4/NF-κB/NLRP3 signaling pathway, reduces inflammatory injury, and relieves inflammation-mediated suppression of erythropoiesis (Tian et al., 2016). In chemotherapy-induced anemia mouse models, SWD significantly increased RBC count and Hb levels (Liu et al., 2018). Pathological analysis further revealed that SWD reduced bone marrow adipocyte proliferation, increased RBC counts, and alleviated bone marrow cell damage in rats with blood deficiency syndrome. Moreover, combined treatment with SWD and EPO was shown to upregulate renal EPO and EPOR expression, inhibit oxidative stress and inflammatory cytokines, and consequently improve both renal function and anemia (Wu et al., 2019). Collectively, SWD ameliorates RA by regulating bone marrow function, promoting EPO expression, inhibiting apoptosis, and enhancing renal EPO mRNA expression.

#### 3.3.5 Yixuesheng capsule

Yixuesheng capsule (YXS) is a proprietary Chinese medicine composed of twenty herbs, including Asini Corii Colla, Astragali Radix, and Polygoni Multiflori Radix Praeparata. It is indicated for invigorating the spleen, nourishing blood, and tonifying the kidney. Asini Corii Colla and Astragali Radix serve as the principal components—promoting erythropoiesis, enhancing energy metabolism and immune function—while Polygoni Multiflori Radix Praeparata, Angelicae Sinensis Radix, and Paeoniae Radix Alba act as secondary components that support renal function, stimulate blood cell production, and stabilize metabolic parameters. Crataegi Fructus, as an auxiliary herb, facilitates digestion and absorption, thereby augmenting overall therapeutic efficacy (Zhang S. N. et al., 2025).

YXS improves RA mainly through anti-inflammatory and iron-regulatory pathways. It reduces pro-inflammatory cytokines (TNF-α, VEGF-A) and suppresses STAT3 signaling, thereby alleviating inflammation-mediated suppression of erythropoiesis and indirectly improving EPO responsiveness (Xu et al., 2023). In iron deficiency anemia models, YXS elevates plasma iron, attenuates inflammatory responses, enhances antioxidant capacity, and reduces oxidative damage to RBCs, acting synergistically with EPO to improve anemia (Zhang S. N. et al., 2025).

#### 3.3.6 Shengxuebao mixture

Shengxuebao mixture (SXBM) is an empirical prescription that has been long used by Chinese clinical experts in TCM. It is composed of seven herbs, including Polygoni Multiflori Radix Praeparata, Ligustri Lucidi Fructus, Mori Fructus, Ecliptae Herba, Paeoniae Radix Alba, Astragali Radix, and Cibotii Rhizoma (Yu et al., 2025). In the prescription, Radix Polygoni Multiflori Preparata is the monarch herb, which has the effect of nourishing the liver and kidneys, cooling the blood, and promoting convergence. Ligustri Lucidi Fructus and Mori Fructus as the ministerial herbs, which, together with the monarch herb, nourish the liver and kidneys. Astragali Radix, as an assistant herb, invigorates energy and blood, strengthens digestive function, and stabilizes immune defense. This prescription further supports the synergistic actions of the monarch and assistant herbs, enhancing their therapeutic effects. The whole recipe is well-composed, with the monarch, minister, assistant, and envoy herbs each fulfilling their respective roles. (Chen X. F. et al., 2024).

SXBM exerts hematopoietic effects by regulating hematopoietic factors and attenuating inflammatory injury to hematopoietic organs. Specifically, it inhibits NF-*κ*B and HIF-1α overactivation, reduces TNF-α levels, and relieves chronic inflammation-mediated suppression of EPO synthesis and erythropoiesis (Yu et al., 2025; Li A. L. et al., 2022). Clinical studies have demonstrated that SXBM significantly improves RA in stage 3–5 CKD patients without dialysis, increasing serum albumin, improving nutritional status, and elevating estimated glomerular filtration rate (eGFR), indicating its renoprotective effect and indirect restoration of endogenous EPO secretion (Wang et al., 2019). In hemodialysis patients, SXBM also corrects anemia, raises Hb and albumin levels, alleviates micro-inflammation, reduces RBC destruction, and promotes renal function recovery, with a favorable safety profile (Xu and Peng, 2021).

#### 3.3.7 Shengxuening tablet

Shengxuening (SXN) tablets are mainly derived from silkworm fecal extract. The preparation process involves extraction, saponification, acidification, washing, and iron complexation to yield sodium iron chlorophyllin and chlorophyll derivatives (Wang et al., 2023). The sodium iron chlorophyllin in SXN is a divalent organic iron complex structurally similar to human heme, conferring higher bioavailability than inorganic oral iron preparations. This medication promotes blood circulation, relieves pain, and enhances hematopoietic function and systemic energy metabolism, effectively alleviating anemia symptoms in patients with RA (Liu et al., 2022).

SXN can inhibit hepcidin expression, regulate iron metabolism homeostasis, increase serum ferritin and transferrin saturation, reduce soluble transferrin receptor level, improve iron storage and transport efficiency, and provide sufficient iron substrate for EPO-mediated erythropoiesis (Mei et al., 2021; Ding et al., 2021). Research indicates that SXN ameliorates iron metabolism in anemic rats, evidenced by elevated serum ferritin and transferrin saturation alongside reduced transferrin and soluble transferrin receptor levels. These adjustments collectively enhance iron storage, transport, and subsequent Hb synthesis (Lei et al., 2016; Zeng et al., 2020). A 2025 meta-analysis of 31 RCTs involving 2,372 patients demonstrated that SXN significantly outperformed placebo and other control drugs in raising Hb levels, serum ferritin, and transferrin saturation, with a superior safety profile (Zheng et al., 2025). The corrective effect of SXN on RA is attributed to the dual action of iron supplementation and internal stimulation by EPO. By utilizing these two pathways, it inhibits the apoptosis of hematopoietic cells and regulates iron homeostasis, thereby achieving the therapeutic effect for RA (Tang et al., 2020).

#### 3.3.8 Fufang E’jiao Jiang

Fufang E’jiao Jiang (FEJ) originates from the classic prescription *Liang Yi Gao*, proposed by Zhang Jiebin during the Ming Dynasty to improve both metabolic support and erythropoiesis. In this formulation, Asini Corii Colla serves as the monarch herb to nourish blood and promote circulation. Codonopsis Radix and Red Ginseng act as minister herbs to enhance systemic energy metabolism, while Rehmanniae Radix Preparata promotes erythropoiesis, provides essential nutrients, and supports bone marrow hematopoiesis. Crataegi Fructus improves gastrointestinal function and addresses digestive stagnation associated with metabolic insufficiency. The combined action of these components achieves coordinated enhancement of both metabolic energy and hematopoietic function (Zhao et al., 2023). Its clinical indications include dizziness, blurred vision, palpitations, insomnia, anorexia, and anemia related to energy deficiency and hematopoietic insufficiency.

FEJ improves RA through the dual regulation of EPO expression and EPOR sensitivity (Chen et al., 2012). In RA rat models, FEJ promotes renal EPO expression and upregulates bone marrow EPOR, thereby enhancing hematopoietic cell responsiveness to endogenous EPO (Liu et al., 2014b). Simultaneously, FEJ improves the bone marrow hematopoietic microenvironment by upregulating IL-1β, IL-3, and granulocyte-macrophage colony-stimulating factor, while suppressing TGF-β expression, collectively promoting erythroid differentiation and proliferation (Liu et al., 2014a).

#### 3.3.9 Shen-Shuai-Ning capsule

Shen-Shuai-Ning (SSN) capsule is formulated with multiple TCM, including Pseudostellaria heterophylla, Coptis chinensis, Pinellia ternata, Tangerine peel, Poria cocos, Rhubarb, Salvia miltiorrhiza, Achyranthes bidentata, Safflower, and Glycyrrhiza uralensis. This prescription is traditionally used for invigorating the spleen and kidney, activating blood circulation and resolving stasis, regulating intestinal motility, and eliminating turbid pathogenicity (Chen X. J. et al., 2018). Among these, Pseudostellaria heterophylla and Poria cocos are responsible for tonifying the spleen and strengthening the kidney; Rhubarb, Coptis chinensis, Pinellia ternata, and Tangerine Peel are involved in promoting bowel movement and eliminating turbidity; Salvia miltiorrhiza, Achyranthes, and safflower are known to promote blood circulation and remove blood stasis, and Glycyrrhiza uralensis is responsible for harmonizing all the medicines. When combined, these medicines synergistically exert therapeutic effects (Ni et al., 2020).

SSN improves RA primarily through anti-inflammatory and renoprotective pathways. It significantly reduces pro-inflammatory cytokines such as TNF-α, IL-1β, and IL-6 by inhibiting NF-*κ*B activation, alleviates renal interstitial inflammation and tubular injury, preserves the number and function of renal EPO-producing cells, and restores endogenous EPO secretion (Ma et al., 2023; Gao and Zhang, 2024). In adenine-induced CKD rat models, SSN significantly improved renal function and anemia, delayed CKD progression, and these effects were attributable to inhibition of renal inflammation and attenuation of renal interstitial fibrosis (Liu Y. et al., 2024).

#### 3.3.10 Huangkui capsule

Huangkui capsule (HKC) is prepared from the flower extract of *Abelmoschus manihot* (L.) Medic. The bioactive constituents of HKC mainly consist of quercetin, isoquercitrin, hyperoside, gossypetin-8-O-β-D-glucuronide, and quercetin-3′-O-glucoside (Cai et al., 2017). These compounds exhibit significant anti-inflammatory, antioxidant, and anti-fibrotic activities, along with potent radical-scavenging activity and renal tubular epithelial repair properties.

HKC exerts renoprotective and anti-anemic effects through anti-inflammatory, antioxidant, and anti-fibrotic actions. It inhibits pro-inflammatory cytokine release, reduces proteinuria and blood urea nitrogen, attenuates glomerular immune responses and renal interstitial inflammation, and preserves renal EPO-secreting function (Xie et al., 2024). In streptozotocin-induced diabetic nephropathy rat models, HKC alleviates renal inflammation and glomerular injury by activating the PPAR pathway, thereby indirectly ameliorating EPO synthesis impairment resulting from renal injury. Additionally, its potent radical-scavenging activity reduces oxidative damage to renal tubular epithelial cells and erythrocytes, prolongs erythrocyte lifespan, and contributes to anemia correction (Ge et al., 2016).

## 4 Conclusions, limitations, and prospects

RA, a common complication of CKD, arises from a multifactorial pathogenesis in which progressive loss of renal function impairs EPO synthesis, while concurrent disruptions in iron metabolism, chronic inflammation, oxidative stress, and uremic toxin accumulation further compromise erythropoiesis. Elevated hepcidin levels, driven by inflammatory mediators such as IL-6 and TNF-α, sequester iron within reticuloendothelial stores, creating a state of functional iron deficiency that exacerbates EPO resistance and anemia severity (Yuan et al., 2024; Orrico et al., 2023). Current therapeutic strategies, including ESAs, HIF-PHIs, iron supplementation, and transfusion, have substantially improved anemia management but face persistent limitations. ESA hyporesponsiveness affects 5%–30% of patients and is associated with increased mortality, while the long-term cardiovascular and oncological safety of HIF-PHIs remains under active debate. Iron overload from supplementation and transfusion poses additional risks, and current approaches often fail to address the underlying inflammatory and metabolic drivers of anemia. In this context, TCM has emerged as a promising adjunctive strategy, with monomers, extracts, and formulations demonstrating potential through three convergent pathways: HIF-2α stabilization to enhance endogenous EPO production, suppression of inflammatory signaling to relieve EPO inhibition, and regulation of iron homeostasis via hepcidin downregulation to improve iron utilization and EPO sensitivity. These multi-target characteristics distinguish TCM from conventional single-agent therapies and position it as a valuable complementary option, particularly for patients with ESA resistance, inflammation-driven anemia, or functional iron deficiency.

A comprehensive synthesis of the available evidence reveals that TCM regulates EPO through an integrated, multi-layered network rather than a single linear pathway. Certain TCM monomers such as luteolin act as direct PHD2 inhibitors, preventing HIF-2α degradation and promoting EPO transcription. Polysaccharides including Angelica sinensis polysaccharide and steamed Panax notoginseng enhance HIF-2α stability and restore renal EPO mRNA expression, while the JPYS decoction activates upstream ERK1/2 signaling to promote HIF-2α accumulation. ASP also activates JAK2/STAT5 and PI3K/Akt pathways to amplify the hematopoietic response to EPO. Simultaneously, multiple components such as rhein, emodin, and the baicalin-geniposide combination suppress NF-*κ*B and reduce IL-6 and TNF-α release, relieving inflammation-mediated suppression of EPO synthesis. Furthermore, silkworm feces extract and the SXN tablet inhibit hepcidin expression through BMP6/SMAD4 and IL-6/STAT3 pathway blockade, enhancing iron availability and EPO sensitivity. When comparing molecular targets across components, a notable convergence on the HIF-2α-EPO axis is observed among luteolin, silkworm feces extract, and ASP, while NF-*κ*B inhibition is shared by rhein, emodin, and tanshinone IIA. However, important differences exist: monomers such as luteolin directly bind PHD2, whereas polysaccharides and formulations act indirectly; most components selectively target HIF-2α, whereas DBD predominantly activates HIF-1α; and iron metabolism is regulated either through hepcidin-ferroportin targeting (silkworm feces extract, SXN) or via inflammation suppression and Nrf2 activation (rhein, catalpol). These target differences suggest that components directly stabilizing HIF-2α may benefit patients with severe EPO deficiency, while those suppressing inflammation or inhibiting hepcidin may be more appropriate for patients with inflammation-driven ESA resistance or functional iron deficiency, and combinations acting on complementary pathways may yield synergistic effects. These mechanistic distinctions and therapeutic implications are systematically summarized in Table 1, which compares the primary molecular targets, pathways, and HIF isoform selectivity of each major TCM component.

Despite the promising potential of TCM, significant limitations must be acknowledged. First, most TCM studies for RA are confined to preclinical models, and their findings have not been translated into clinical practice. For compounds such as rhein and tanshinone IIA, research has focused on anti-fibrotic and anti-inflammatory effects rather than direct EPO assessment, leaving an incomplete evidence chain (Yang et al., 2018). Second, the safety of TCM in renal insufficiency warrants consideration: impaired filtration prolongs herbal metabolite half-lives, increasing accumulation and toxicity risk; many components are renally excreted, and even safe herbs may pose risks when kidney function declines. Herb-drug interactions via CYP450 enzymes (CYP3A4, CYP2D6, CYP2C9) and P-glycoprotein may alter the pharmacokinetics of co-administered ESAs, HIF-PHIs, iron preparations, and antihypertensives, necessitating dose adjustment, patient education, monitoring, and pharmacokinetic studies. Third, most TCM formulas have complex metabolism, low bioavailability, and altered pharmacokinetics in CKD, leading to unstable efficacy (Chen, 2024); molecular targets are often inferred from indirect assays rather than identified by advanced techniques such as chemical proteomics, CETSA, or CRISPR screens. Fourth, most TCM studies lack rigorous RCTs with adequate sample sizes and long-term follow-up; high-quality, multicenter trials with standardized outcomes are urgently needed.

Future research on RA should address mechanistic and translational gaps through several priority areas centered on EPO regulation. In this regard, given the multi-target potential of TCM, network pharmacology and metabolomics should be employed to identify compounds that directly modulate the HIF-2α/EPO axis and its upstream regulators; for example, Astragaloside IV may enhance EPO transcription via HIF pathway activation, a hypothesis that requires validation through gene knockout and reporter assays, while optimized delivery systems such as nanoparticle carriers could further improve the bioavailability of EPO-modulating TCM components, with reported benefits on intestinal iron absorption and gastrointestinal tolerance (Sun K. et al., 2022; Portolés et al., 2021). Beyond TCM, emerging strategies targeting the HIF pathway, the hepcidin-ferroportin axis, and inflammatory signaling offer complementary approaches to augment EPO action, including small-molecule hepcidin inhibitors (e.g., monoclonal antibodies or siRNA) and ferroportin agonists that can correct functional iron deficiency and thereby enhance EPO responsiveness (Carullo et al., 2024), as well as nano-iron formulations such as iron carboxymaltose that provide high bioavailability with reduced oxidative stress to support EPO-driven erythropoiesis; modulation of the gut microbiota-iron axis may also improve iron availability and reduce inflammation via short-chain fatty acids and bile acid regulation (Mann et al., 2024), and monoclonal antibodies against IL-6, TNF-α, and other pro-inflammatory cytokines, along with JAK/STAT inhibitors, could relieve inflammation-induced suppression of EPO synthesis and signaling. Additionally, although gut microbiota dysbiosis has been linked to RA, causal relationships remain unclear, and future studies using gnotobiotic models and fecal microbiota transplantation are needed to determine whether microbiota modulation directly influences EPO production or erythropoietic efficiency. In parallel, hepcidin antagonists and HIF-PHIs require comparative effectiveness trials against current standard therapies across different CKD stages, with careful monitoring of long-term cardiovascular and oncological safety given their direct impact on the EPO pathway. Finally, precision medicine should move beyond uniform Hb targets toward integrated multi-omics signatures that incorporate genetic, inflammatory, metabolic, and microbial parameters; such stratification could guide individualized EPO-based therapies, with EPOR gene polymorphisms, HIF pathway variants, and iron metabolism-related SNPs (e.g., TMPRSS6) helping to predict patient responses to HIF-PHIs or hepcidin inhibitors, while artificial intelligence-assisted drug repositioning may further accelerate the discovery of novel EPO-regulatory targets (Wang X. et al., 2023).

This review elucidates the multifactorial pathogenesis of RA and redefines the therapeutic landscape by integrating evidence-based TCM interventions with conventional treatment modalities. Despite promising preclinical data, substantial challenges remain in translating these findings into clinical practice, and the efficacy and safety of TCM for RA warrant further validation through well-designed studies. Future advances in this field are expected to facilitate a paradigm shift from symptom-oriented management toward multidimensional pathophysiology-targeted therapy, ultimately improving the quality of life of patients with CKD and promoting the rational integration of TCM into routine clinical practice.
